# Model Vestibular Nuclei Neurons Can Exhibit a Boosting Nonlinearity Due to an Adaptation Current Regulated by Spike-Triggered Calcium and Calcium-Activated Potassium Channels

**DOI:** 10.1371/journal.pone.0159300

**Published:** 2016-07-18

**Authors:** Adam D. Schneider

**Affiliations:** Physics, McGill University, Montreal, Quebec, Canada; College de France, FRANCE

## Abstract

*In vitro* studies have previously found a class of vestibular nuclei neurons to exhibit a bidirectional afterhyperpolarization (AHP) in their membrane potential, due to calcium and calcium-activated potassium conductances. More recently *in vivo* studies of such vestibular neurons were found to exhibit a boosting nonlinearity in their input-output tuning curves. In this paper, a Hodgkin-Huxley (HH) type neuron model, originally developed to reproduce the *in vitro* AHP, is shown to produce a boosting nonlinearity similar to that seen *in vivo* for increased the calcium conductance. Indicative of a bifurcation, the HH model is reduced to a generalized integrate-and-fire (IF) model that preserves the bifurcation structure and boosting nonliearity. By then projecting the neuron model’s phase space trajectories into 2D, the underlying geometric mechanism relating the AHP and boosting nonlinearity is revealed. Further simplifications and approximations are made to derive analytic expressions for the steady steady state firing rate as a function of bias current, *μ*, as well as the gain (i.e. its slope) and the position of its peak at *μ* = μ*. Finally, although the boosting nonlinearity has not yet been experimentally observed *in vitro*, testable predictions indicate how it might be found.

## Introduction

A primary goal of computational neuroscience is to understand the nature of the “neural code” with which sensory information is represented and processed by successive stages of neurons in the nervous system. Sensory neurons were first shown to encode stimulus features such as intensity, in the rate at which they fire action potentials. Accordingly, sensory neurons are often characterized by “tuning curves”, which provide a map from a particular stimulus parameter (such as intensity) to the neurons output firing rate [1]. Although linear transformations are known to preserve information, nonlinear transformations are essential for the selective coding of particular stimulus features, as well as using a neurons full information transmission capacity [2]. In the vestibular system, for example, semicircular canal afferents have long been known to primarily encode angular head velocity through firing rate modulations that vary linearly with increasing stimulus amplitude until saturation or rectification occurs [3], whereupon the neuron has reached its maximum or minimum firing rate, respectively. More recently however, *in vivo* studies have shown that neurons in the medial vestibular nuclei (VN) exhibit a boosting nonlinearity in their input-output tuning curves (i.e. firing rate output, versus stimulus, afferent, or bias current input; a.k.a. tuning or f-I curve) [4]. This boosting nonlinearity is characterized by a linear region with a small positive slope for low afferent input currents, and a linear region with higher positive slope for larger afferent inputs, rather than the more common occurrence of a higher slope at low bias currents. *In vitro* studies, on the other hand, measure the membrane potential time course and have developed a conductance based Hodgkin-Huxley-type VN model, with voltage-activated calcium and calcium-activated potassium channels that produce a specific bidirectional afterhyperpolarization (AHP) [5, 6]. In this paper, a simpler version of this model is shown to produce a boosting nonlinearity similar to that observed experimentally *in vivo* [4], for increased calcium conductances, *g*_*Ca*_, which acts as a bifurcation parameter. In order to shed some light on the underlying mechanisms responsible, a simplified integrate-and-fire (IF) type model is created that is more analytically tractable but preserves the bifurcation structure and boosting nonlinearity under investigation.

It requires a system with at least two variables with nonlinear dynamics to produce action potentials with sodium and potassium currents; the simplicity of IF models is that they replace these spike generating ion channels, with a simpler boundary condition that takes the voltage from threshold back to a reset value [7]. Single variable (i.e. membrane voltage, V) IF models can then be made to have more realistic subthreshold dynamics (which will be required to produce the AHP) by adding back a voltage dependent function, *ψ*(*V*). A *linear* “leak” term (giving an LIF) allows the membrane to return to a given resting potential in the absence of stimulation, and a *quadratic* term (giving a QIF) will also add a depolarizing up stroke in the voltage preceding action potentials to better match their shape. A combined linear and exponential function (giving an EIF), has been shown to better fit experimental data [8–10], at a sacrifice to its analytic tractability. Such IF models can be further generalized to include any extra currents, which may require additional dynamic gating variables, such as spike-triggered adaptation currents (often denoted by W) which serve to decrease V. However, such additional variables also require additional reset conditions, for the change in W upon spiking. The spiking dynamics of such 2-variable (i.e. V,W) adaptive IF models have been extensively studied [11–13], showing that they can produce a variety of spiking behaviors including a similar boosting nonlinearity and a unidirectional AHP [14], for certain parameter combinations.

In this paper, a Hodgkin Huxley (HH) type spiking VN neuron model is reduced to a QIF model generalized to include the calcium and calcium-activated potassium currents, which preserves the bifurcation structureand the boosting nonlinearity observed in the original HH model. The spiking trajectories of the resulting 3-variable adaptive QIF model are then projected into the 2D V-W phase space, revealing an intuitive geometrical picture linking the AHP phase space trajectories with the low gain region of the boosting nonlinearity. Simplifying the models reset conditions and making some additional assumptions, allows for an analytic approximation for the steady state firing rate and its gain (i.e. f-I curve slope) across a similar boosting nonlinearity, as well as the bias current at which the gain is peaked, *μ* = μ*. Although this boosting nonlinearity in the f-I curve of VN neurons has not be experimentally observed *in vitro*, the link with the AHP generation provides the testable prediction that it should be found in the transition to increased bias currents where the AHP no longer occurs.

## Results

### HH model produces boosting nonlinearity with AHP and bifurcation through bursting separating low and high gain regions

**Fig 1:**
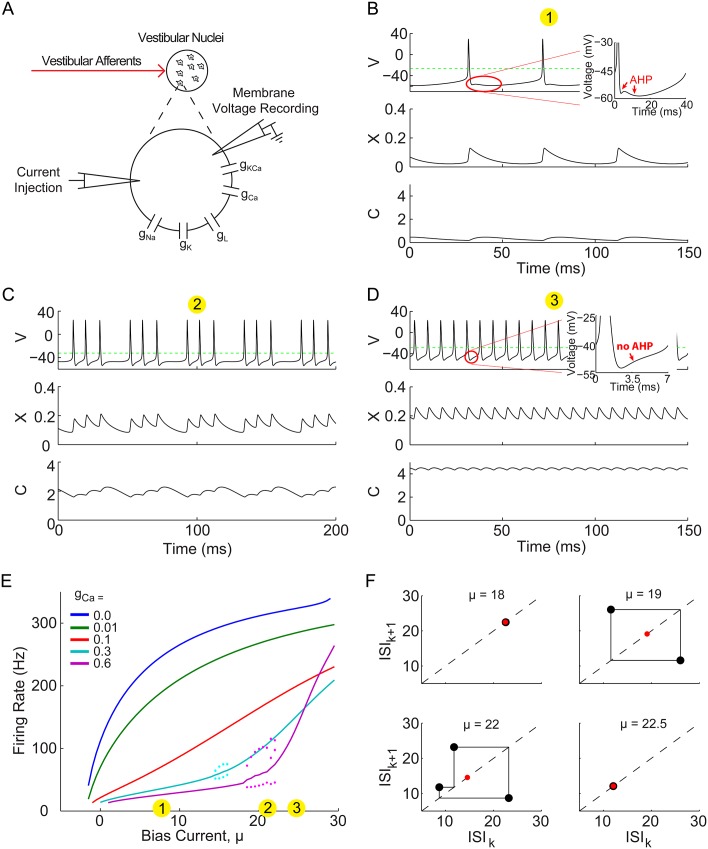
Calium and calcium-activated potassium currents induce boosting nonlinearity, AHP, and bifurcation through bursting. (A) A schematic indicating that the neuron model of a vestibular nuclei neuron with conductance based ion channels as described in Models and Methods Eq (5), as well as a constant current injection, which drive the membrane voltage, the “recorded” model output, to generate action potentials. (B-D) Example time series of the simulated membrane voltage, with calcium gating variable, *x*, and calcium concentration, *C*, below. Insets show zoom of region preceding spikes either with or without an AHP. Dashed green lines indicate the voltage threshold at which spike times are said to occur. Examples correspond to *g*_*Ca*_ = 0.6, for the bias current values indicated by the numbered yellow circles in panel E. (E) The firing rate as a function of constant bias current injection, or “f-I curve”. Colored lines correspond to the average 1/ISIs for the calcium conductance values indicated, with the colored dots indicating each 1/ISI value of the bursting solutions (i.e. panel C). (F) ISI return maps for four example bias currents with *g*_*Ca*_ = 0.6, showing how the stable limit cycle (*μ* = 18) destabilizes into stable 2-spk bursting (*μ* = 19), and then 3-spk bursting (*μ* = 22), and back to a stable single spike limit cycle (*μ* = 22.5). Red dots indicate the mean ISI.

Fig 1A shows a schematic of the conductance based Hodgkin-Huxley (HH) type model (defined by Eqs (4) and (5) in Models and Methods), which was simulated with different calcium conductance strengths, *g*_*Ca*_, over a range of constant bias current injections, *μ*. Although the full HH model has 4 dynamical variables, example traces of the voltage, *V*, as well as the gating variable, *x*, and calcium concentration, *C*, are shown for different bias currents, and a specific calcium conductance in Fig 1B–1D. The dashed green lines indicate a voltage threshold, crossings of which are defined to be spike times, which in turn define a sequence of inter-spike-intervals (ISIs). In panels B and D, red circles indicate regions immediately after spiking that are shown in insets, indicating that the specific AHP in which the voltage changes directions twice, occurs at low bias but not high bias currents. At each bias current value, 1/ISI can be used to give the firing rate, which can be averaged over possibly different ISIs in the case of bursting solutions, such as shown in Fig 1C. These average firing rates are plotted as a function of bias current (known as an f-I curve) in Fig 1E, also with the individual 1/ISIs of the bursts as dots. A boosting nonlinearity (i.e. an increase in gain with an increase in bias current) can be seen to occur for the two highest *g*_*Ca*_ vales (cyan and magenta curves), while for the intermediate *g*_*Ca*_ value (red curve) the effect is to linearize the f-I curve by reducing the gain at the onset of spiking near *μ* = 0.

It can also be seen that when the boosting nonlinearity occurs, stable limit cycles of a single ISI are present for sufficiently low or high bias currents, while stable bursting limit cycles (i.e. 2-spk burst, 3-spk burst) appear for intermediate bias current values where the gain (i.e. f-I slope) changes across the boosting nonlinearity. This bifurcation through bursting is characterized by plotting ISI return maps at various bias currents across the bursting region, as are shown in Fig 1F for *g*_*Ca*_ = 0.6. From the top right panel stable 2-spk bursting can be seen to transition to stable 3-spk bursting in the lower left panel, before returning to a stable 1-spk limit cycle at higher biases. This appears to be a global “period adding” bifurcation through bursting, however, analysis of the bursting mechanism is beyond the scope of this paper which aims to understand the change in gain across the boosting nonlinearity. In order to simplify the model and isolate the mechanism underlying this boosting nonlinearity, this HH model was reduced to an analytically tractable integrate-and-fire (IF) type model, which preserves both the boosting nonlinearity and period adding bifurcation.

### QIF reduction of HH model can preserve subthreshold bifurcation structure, boosting nonlinearity, and bifurcation through bursting

**Fig 2:**
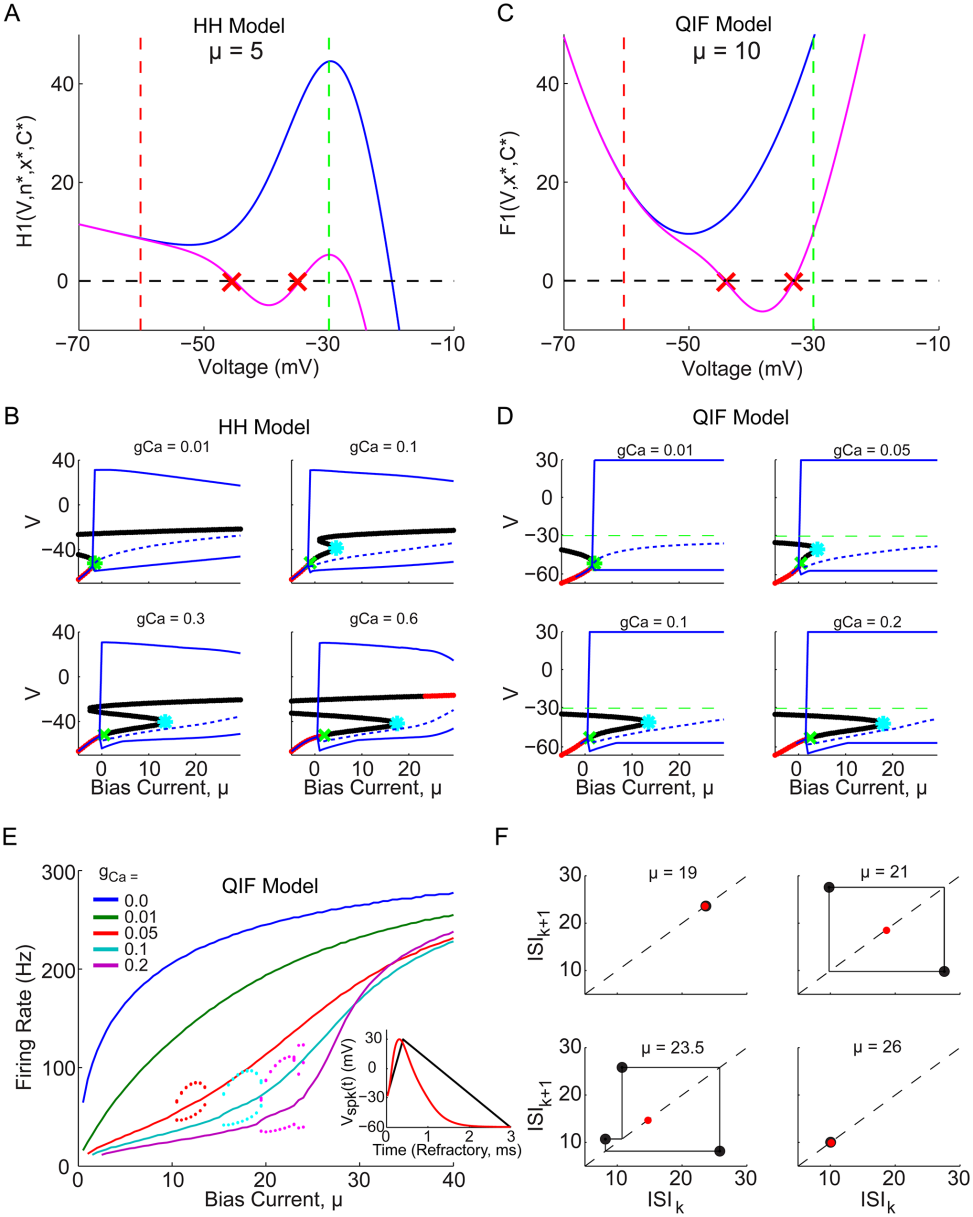
Reduced QIF model captures subthreshold bifurcation structure, boosting nonlinearity, and bursting bifurcation of HH model. (A) *H*_1_(*V*, n*, x*, C*) is plotted for *μ* = 5, showing how there are either 0 or 2 subthreshold (between the green and red dashed lines) fixed points (i.e. zero crossings), for the *g*_*Ca*_ = 0 and *g*_*Ca*_ = 0.6 cases, respectively. (B) Bifurcation diagram for HH model at four different values of *g*_*Ca*_ as indicated. Fixed points at each bias current value correspond to zeros of *H*_1_(*V*, n*, x*, C*), with red indicating stable and black indicating unstable. A green star indicates a saddle-node bifurcation, while a green x indicates an Hopf bifurcation, and a cyan star indicates the annihilation point of the two remaining unstable fixed points. Dashed blue lines indicate the mean voltage, while solid blue lines indicate its maximum and minimum. (C) shows the equivalent *F*_1_(*V*, x*, C*) for the reduced QIF model, for the *g*_*Ca*_ = 0 and *g*_*Ca*_ = 0.6 cases, also indicating the two subthreshold fixed points. Green and red dashed lines indicate the voltage threshold and reset, respectively, indicating that the two functions have the same concave shape needed to generate the same subthreshold bifurcation structure. (D) Bifurcation diagram for QIF model at four different values of *g*_*Ca*_ as indicated, with fixed points now corresponding to the zeros of *F*_1_(*V*, x*, C*), and additional dashed green lines indicating the voltage threshold. (E) The QIF model f-I curve, with colored lines and dots as in Fig 1E. Inset shows artificial piecewise linear action potential used to simulate refractory period (black), with an example HH model action potential superimposed (red) for comparison. (F) ISI return maps for four example bias currents with *g*_*Ca*_ = 0.2, showing how the stable limit cycle undergoes the same period adding bifurcations through bursting as the HH model (see Fig 1F), in the region which separates the low and high gain regions of stable 1-spk firing.

To understand the mechanism underlying the HH model’s boosting nonlinearity, a reduced integrate-and-fire (IF) type model is generated, that is analytically tractable yet preserves the boosting nonlinearity and underlying bifurcation structure. This was done by replacing the gating variable, *n*, and related spike generating currents by a nonlinear function, *ψ*(*V*), with an additional voltage threshold and reset mechanism, as described in Models and Methods. The model’s bifurcation structure can be found by calculating the fixed points at each different bias current, which are defined by the zeros of the function *H*_1_(*V*, n*, x*, C*) (see Models and Methods Eq (6)). This function is plotted in Fig 2A for *g*_*Ca*_ = 0 and *g*_*Ca*_ = 0.6, at an example bias current *μ* = 5. The green dashed line indicates the voltage threshold used in Fig 1B–1D to define the spike times, and the red dashed line indicates the voltage reset value that will be used, which roughly corresponds to the minimum voltage during the action potential in the voltage time series in Fig 1B–1D. These curves are shifted up and down with *μ* and the zero crossings correspond to the HH model’s fixed points, with their stability calculated via Eq (7). For sufficiently low bias currents, there are three fixed points and the system does not spike spontaneously. As *μ* is increased the curve is shifted upwards and eventually the two lower fixed points annihilate, generally resulting in the onset of spiking via a saddle-node bifurcation. However, its is possible for spiking to begin via a Hopf bifurcation, before the two subthreshold fixed points have been annihilated. The fixed point bifurcation diagrams are plotted as a function of bias current for each of the non-zero calcium conductances in Fig 2B, with red dots indicating stable fixed points, and black dots indicating unstable fixed points. The blue lines indicate the maximum and minimum values of the spiking limit cycles, and the onset bifurcation is indicated by a green star for a saddle-node and a green x for an Hopf bifurcation. In the case of the Hopf, the point at which the two remaining unstable subthreshold fixed points annihilate is indicated by cyan stars, which can also be seen to roughly coincide with the region of the bursting solutions. It would appear that the bursting and boosting nonlinearity are closely related to the subthreshold fixed point bifurcation structure, which should be preserved in a reduced IF model.

Although and exponential-IF (EIF) model could provide a better fit to *H*_1_(*V*, n*, x*, C*) in the subthreshold region indicated in Fig 2A, a quadratic-IF (QIF) captures the essential local minimum between threshold and reset necessary to reproduce the two subthreshold fixed points, and has the advantage of being analytically tractable. Although a cubic term could reproduce the entire ‘S’ shape and high voltage FP, it lies above the voltage threshold and can be ignored for our purposes. The two QIF model parameters, *g*_2_ and *V*_2_, can be related to the HH model parameters by linearizing the nonlinear functions in Eq (6) and keeping only terms to second order in *V*, as in Eq (12). However, simply choosing values of *g*_2_ = 0.1 and *V*_2_ = −50 provides a sufficiently good approximation to reproduce the desired phenomena, as can be seen from the resulting function *F*_1_(*V*, x*, C*) Eq (10) plotted in Fig 2C, and bifurcation diagrams in Fig 2D.

The QIF model, defined by Eq (8) in Models and Methods, additionally requires an artificial spike waveform to activate the calcium current gating variable, *x*, as described by Eq (9). The resulting f-I curves for this QIF model are shown in Fig 2E, and can be seen to exhibit the desired boosting nonlinearities, as well as the bursting, similarly to the HH model (although for slightly different values of *g*_*Ca*_). In addition, this QIF model exhibits the same period adding bifurcation through bursting as the HH model, as shown by the QIF models ISI return maps (compare Figs 2F and 1F). Although the QIF model reproduces the boosting nonlinearity, it also reproduces the same bursting patterns; does this mean that the bursting is necessary to create a boosting nonlinearity?

### Does boosting require bursting?

**Fig 3:**
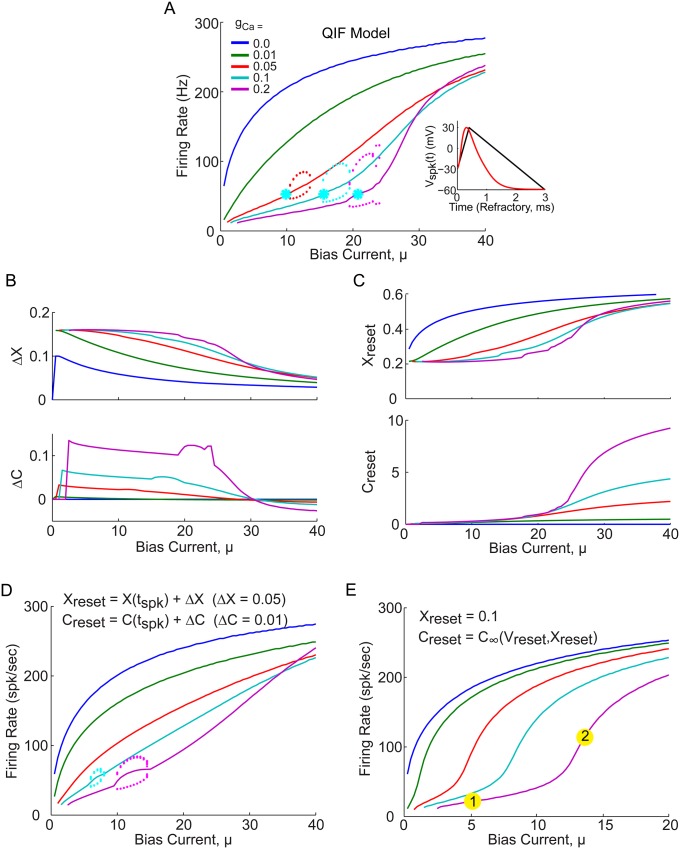
Boosting nonlinearity and bursting depend on reset boundary conditions, not only subthreshold bifurcation structure. (A) Firing rate versus *μ* for the QIF model used in Fig 2E, again with the inset indicating the piecewise linear spike waveform used (black), superimposed with an actual HH model spike (red). Red Xs indicate the bifurcation point of the two subthreshold fixed points which appear to correspond with the onset of bursting. (B) The average Δ*x* and Δ*C* values generated by the spike shape in A, which change significantly with bias current, *μ*. (C) The average *x*_*reset*_ and *C*_*reset*_ values generated by the spike shape in A, also change significantly with bias current. (D) QIF model simulated f-I with fixed Δ*x* and Δ*C* values for all *μ*. (E) QIF model simulated f-I with fixed *x*_*reset*_ and *C*_*reset*_ values, indicates that the boosting nonlinearity can still occur, without any bursting, seemingly independently of the subthreshold bifurcation points, which are the same as in A. Numbered yellow dots indicate examples of low and high gain regions of interest due to boosting nonlinearity most similar in shape to purple f-I curve in panel A.

It would appear the the boosting nonlinearity and bursting, depend intimately on the underlying subthreshold bifurcation which occurs near the onset of bursting (see Fig 3A, red Xs). However, these results actually depend significantly on the artificial spike shape used, which defines the reset conditions but does not effect the subthreshold bifurcation structure shown in Fig 2D. The resulting reset condition can be thought of as the amount by which the gating variables change, Δ*x* and Δ*C*, plotted in Fig 3B, or the reset values themselves, *x*_*reset*_ and *C*_*reset*_, plotted in Fig 3C. Are the changes in these reset values with bias current in fact necessary for the model to produce the boosting nonlinearity or bursting? This question can be answered by simplifying the QIF model in these two different ways, choosing fixed values for Δ*x* and Δ*C*, *or* for *x*_*reset*_ and *C*_*reset*_. The resulting f-I curves for each case are shown in Fig 3D and 3E. In both cases some degree of the boosting nonlinearity can be seen, with a similar bursting occurring in D, but not in E, confirming that one can in fact achieve boosting without bursting.

To understand what is going on, one can think of the QIF model’s 3D phase space in *V*, *x*, and *C*. The voltage is bound by the reset and threshold, starting at *V*_*reset*_ with particular *x*_*reset*_ and *C*_*reset*_ values, and evolving in time until it reaches *V*_*th*_. The possible trajectories through this 3D phase space cannot intersect, and are all defined by the system of Eq (8), which also defines the subthreshold bifurcation structure. It is how the gating variables are reset that regulates bursting; if the gating variables are changed by a fixed amount at reset, then they must also change by an equal and opposite amount during their phase space trajectory in order to be reset back onto the same trajectory. Otherwise, if the gating variables change by a different amount than the reset, a different trajectory through phase space will be selected, resulting in a different ISI. For fixed gating variable resets, however, the phase space trajectory doesn’t matter, the gating variables are always reset to the same values, resulting in the same phase space trajectory and ISI, making bursting impossible.

Although neither of these simplified QIF models capture the physiological realism of the QIF with the artificial spike, they do disentangle the relationship between the boosting nonlinearity, subthreshold bifurcation structure, and bifurcation through bursting. Furthermore, the simplified model in Fig 3E is analytically tractable and will provide a basis for later understanding the model with spike generated reset conditions.

### Analytic firing rate and gain curves for the QIF model, with fixed gating variable reset

To get an analytic expression for the f-I curve and its gain across the boosting nonlinearity, the simplified QIF model with fixed gating variable reset conditions shown in Fig 3E was first considered. As the QIF model is still nonlinear with three dynamic variables, some additional assumptions are needed. Since the original spike generating sodium and potassium channels’ gating variable, *n*, has an average time scale much faster than the additional calcium-related gating variables, *x* and *C*, (*τ*_*n*_ ≈ 1.5*ms* < *τ*_*x*_ = 10*ms*, *τ*_*C*_ = 20*ms*) it can be assumed that the additional gating variables, *x* and *C*, are slow compared to the membrane voltage, *V*. From this they can be set to their mean values which must equal their reset values: *x* = 〈*x*〉 = *x*_*reset*_, and *C* = 〈*C*〉 = *C*_*reset*_, with $\dot{x}=\dot{C}=0$. Although this cannot be true during an AHP in which the voltage changes directions and $\dot{V}=0$ momentarily, it does provide a useful starting place: it reduces the model to a single differential equation in *V* that can be solved analytically Eq (14), where the additional calcium and calcium-activated potassium currents have been redefined as a single mean adaptation current, $\bar{W}\left(V\right)$, defined by Eq (13).

**Fig 4:**
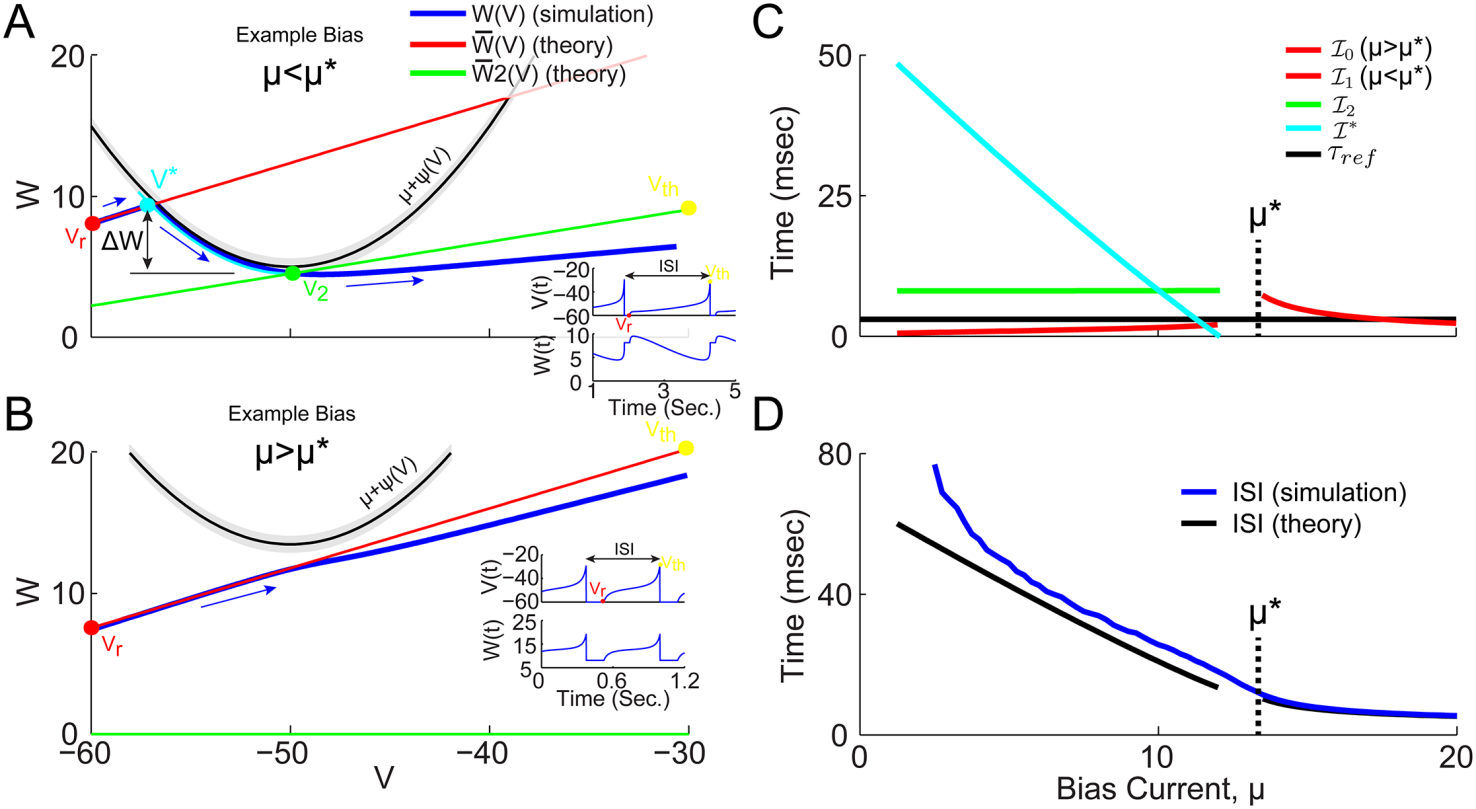
WV phase space projection of trajectories at low and high gain *μ* values, for QIF with *X*_*r*_ = 0.1 and *C*_*r*_ = *C*_∞_(*V*_*r*_, *X*_*r*_)(see Fig 3E) (A) Example phase space trajectory at bias current, *μ* < μ*, for *g*_*Ca*_ = 0.2. Simulated trajectory projected into VW phase space (blue) has blue arrows indicating direction of motion in time. The theoretically predicted trajectory starts at the reset voltage, *V*_*reset*_ (red dot), and travels along $\bar{W}\left(V\right)$ (red line) until it intersects *μ* + *ψ*(V*) (black line) at V* (cyan dot). The grey band indicates the region of $|\dot{V}|<\epsilon$. At this point, the *x* and *C* variable are free to decay (cyan curve) until *W* reaches *V*_2_. Then *x* and *C* are again fixed and give rise to a new mean adaptation current ${\bar{W}}^{*}\left(V\right)$ (see Eq (26), green line) connecting the point *V*_2_ (green dot) and the threshold voltage (yellow dot). Furthermore, the change in *W* between V* and *V*_2_ is assumed to be $\Delta W=\bar{W}\left(V^{*}\right)-\left(\mu -\epsilon \right)$ as indicated (and assumed by Eq (23)). Insets show *V*(*t*) and *W*(*t*) as time series. (B) Example trajectory at bias current, *μ* > μ*, for *g*_*Ca*_ = 0.2. Now $\bar{W}\left(V\right)$ (red line) does not intersect *μ* + *ψ*(*V*), and directly connects *V*_*r*_ (red dot) and *V*_*th*_ (yellow dot). (C) The time intervals for the different trajectory components are plotted as a function of bias current (for *g*_*Ca*_ = 0.2), using colors that match the trajectory components color in (A) and (B). The vertical dashed line indicates μ*. (D) The sum of all the predicted intervals shown in (C) results in the predicted ISI (black), and the average ISI of the simulated data (blue) show how the total interval is dominated by $I^{*}$ when *μ* < μ*.

For this slow gating variable assumption to hold requires that $\dot{V}>\dot{x}=\dot{C}\approx 0$, which is true as long as the depolarizing “spike generating” current, *ψ*(*V*) is greater than the hyperpolarizing adaptation current, $\bar{W}\left(V\right)$ (i.e. $\mu +\psi \left(V\right)>\bar{W}\left(V\right)$, which must be true for sufficiently large *μ*). This results in a 2D VW phase space, in which the depolarizing current, *μ* + *ψ*(*V*) is parabolic, and the hyperpolarizing adaptation current, $\bar{W}\left(V\right)$ is linear. As such, μ* can be defined Eq (15) so that if *μ* < μ* then *ψ* and $\bar{W}$ intersect, while if *μ* > μ* they do not (see Figs 4A and 1B). The simulated trajectories through phase space are superimposed in blue, with blue arrows indicating the direction of the flow from reset to threshold. As long as *μ* > μ* the slow gating variable approximation holds and Eq (14) can be integrated from *V*_*r*_ to *V*_*th*_, resulting in the time interval $I_{0}$ Eq (17). The approximate trajectory from *V*_*r*_ to *V*_*th*_ is superimposed (red curve, $\bar{W}\left(V\right)$) in Fig 4B.

For *μ* < μ*, on the other hand, the linear trajectory starting at *V*_*r*_ can be seen to intersect the parabola at a point denoted V*, defined by Eq (18). The *ϵ* ensures that *F*(*V*) > 0 between *V*_*r*_ and V* and can be integrated to give $I_{1}$ Eq (19). At V*, then *F*(*V*) → 0 and the voltage would come to rest at a fixed point if the slow gating variable assumption *was not* violated; instead the trajectory is now driven by the gating variable dynamics defined by Eq (8b) and (8c). If the trajectory were to cross above the parabola, *μ* + *ψ*(*V*), then *F*(*V*) < 0 and the voltage would have to decrease until it crosses back; so the only way for the voltage to increase up to threshold, is ultimately by following along under the parabola until it reaches the bottom (located at *V* = *V*_2_, *W* = *μ* − *ϵ*) where it is free to increase to threshold. The time interval, $I^{*}$, for the voltage to travel from V* to *V*_2_, is calculated in Models and Methods by allowing the gating variables to change and estimating the time for W* to decay down to *μ* + *ψ*(*V*) (see Eqs (20)–(25)). In the final segment from *V*_2_ to *V*_*th*_ the gating variables are again fixed to their new values, which result in a new ${\bar{W}}_{2}$ value and the green linear trajectory shown in Fig 4A, resulting in the interval $I_{2}$ Eq (27).

Each of these times is calculated for all values of *μ* and are plotted in Fig 4C. The summed times then give the total ISI, which was compared to the simulated ISIs in Fig 4D. It is clear that at low bias current values the intervals are dominated by $I^{*}$ which also has the most significantly nonzero slope. When inverted, the combined intervals defined by Eqs (17), (19), (25) and (27), give an approximation to the steady state firing rate as a function of bias current:

$$\begin{array}{ccc} R\left(\mu \right)=\frac{1}{I_{1}+I^{*}+I_{2}+\tau_{r}} & & \text{,for}\;\mu <\mu^{*}\\ \frac{1}{I_{0}+\tau_{r}} & & \text{,}\;\text{for}\;\mu >\mu^{*} \end{array}$$ (1)

**Fig 5:**
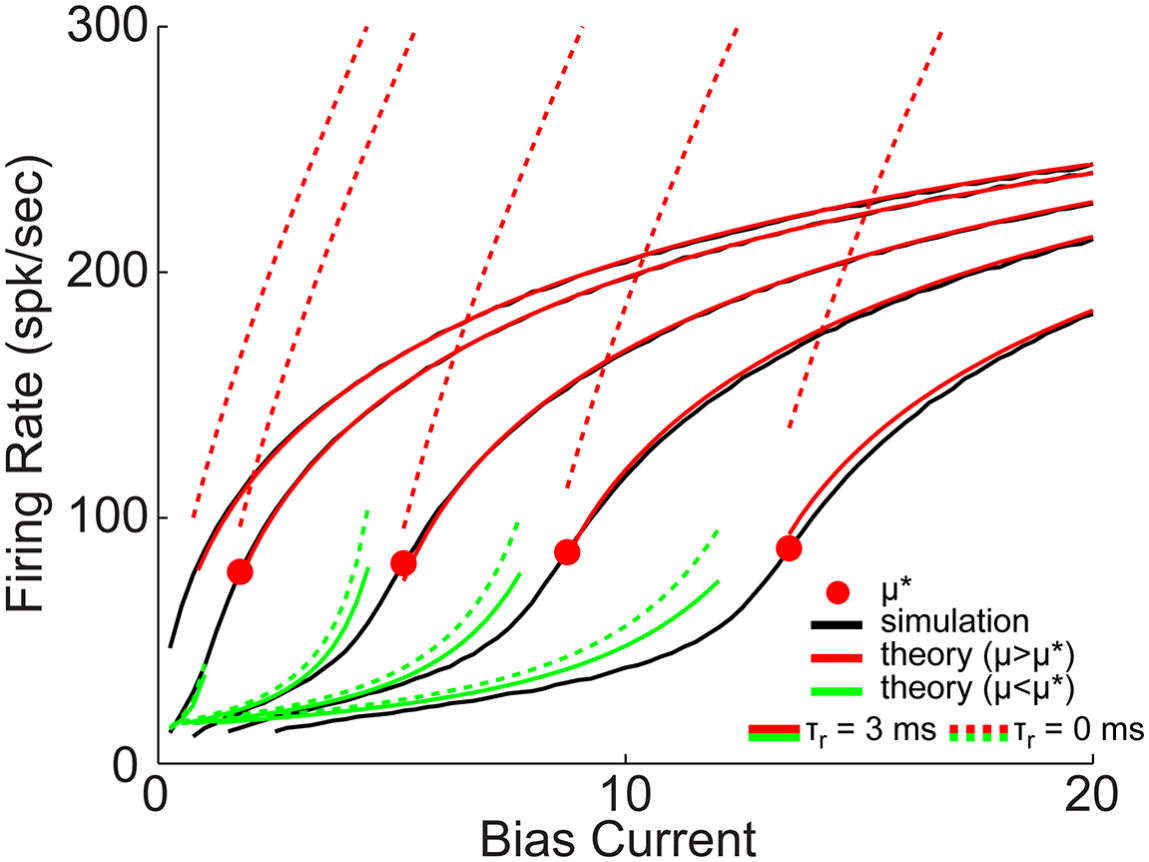
Steady state firing rate, simulations and theory: QIF with *X*_*reset*_ = 0.1 and *C*_*reset*_ = *C*_∞_(*V*_*reset*_, *X*_*reset*_). The firing rate is plotted for the numerical simulations in black, with analytic Eq (1) in green and red, with solid for *τ*_*r*_ = 3 ms, and dashed for *τ*_*r*_ = 0 ms.

Eq (1) is plotted in Fig 5, for both *τ*_*r*_ = 3 ms (solid green and red) as well as *τ*_*r*_ = 0 ms (dashed green and red), superimposed with the firing rate calculated by numerically simulating model Eq (8) directly (black). The solid red dots indicate μ* which can be seen to clearly match the region where the slope of the f-I curves are greatest. For *μ* > μ* the solid red curves are in very good agreement with the black curve, and the saturation (or reduction in slope) can be seen to be due primarily to the refractory period, *τ*_*r*_ (compare solid and dashed red). *μ* < μ* the solid green curves are in reasonable agreement with the black curves (considering the additional approximations needed) at least exhibiting the boosting nonlinearity effect, which does not change significantly with *τ*_*r*_.

**Fig 6:**
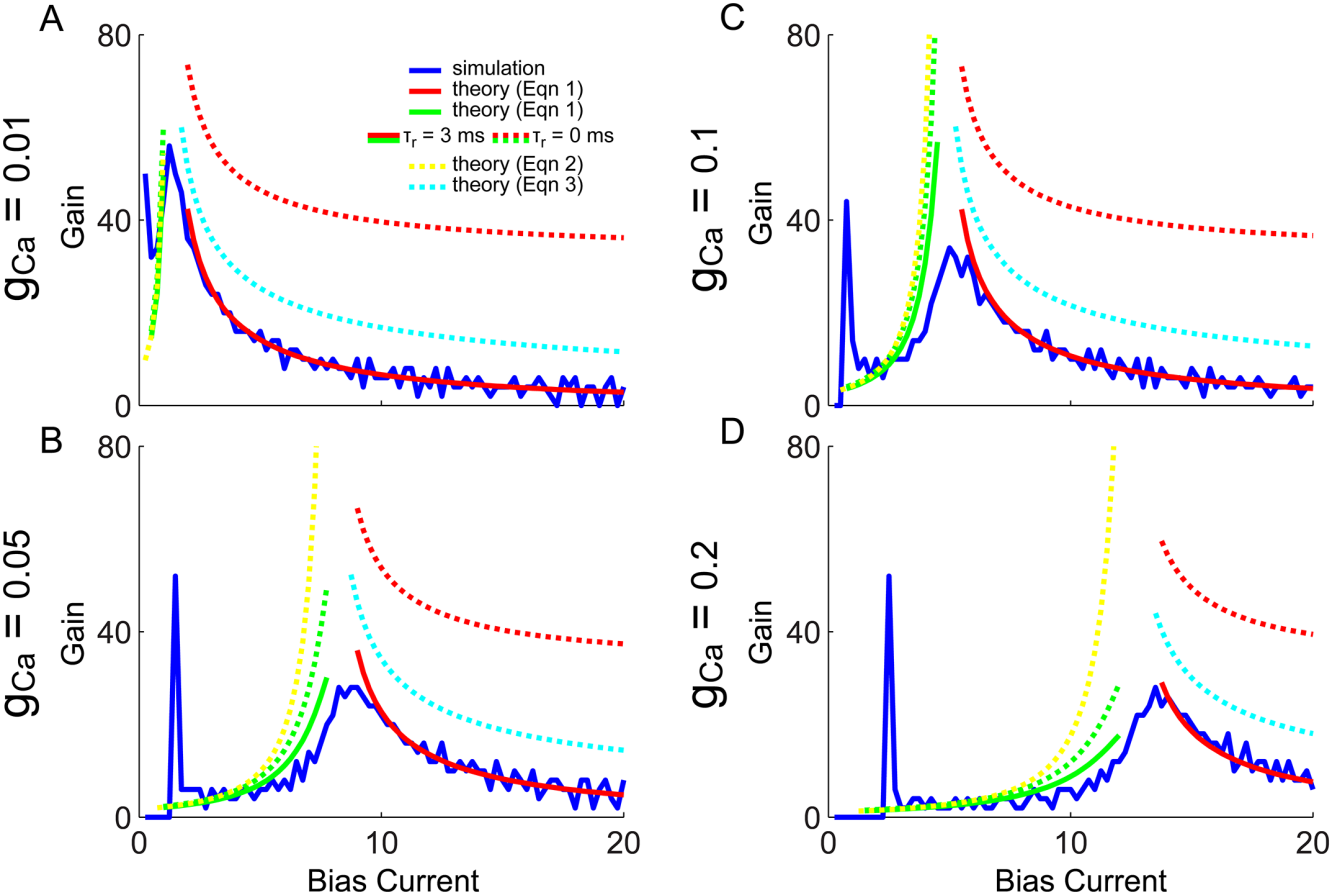
f-I curve gain, simulations and theory: QIF with *X*_*reset*_ = 0.1 and *C*_*reset*_ = *C*_∞_(*V*_*reset*_, *X*_*reset*_). The gain (or f-I curve slope) is plotted for the numerically simulated model in blue, with the derivative of Eq (1) in green and red (solid and dashed). Additionally the approximate gain Eqs (2) and (3) are superimposed in yellow and cyan dashed lines. The peak of the blue gain curve occurs at μ* (vertical black line) where the theoretical predictions all diverge. Panels A-D correspond to increasing values of *g*_*Ca*_.

The gain, or slope of the f-I curves, can next be calculated by simply differentiating Eq (1) with respect to bias current, *G*(*μ*) = ∂*R*(*μ*)/∂*μ*, which is plotted in Fig 6 (green and red solid and dashed curves) for comparison with that calculated from the simulated f-I curves (blue). To derive a more intuitive approximate equation for the gain, the refractory period can be set to zero, *τ*_*r*_ = 0:

$$\begin{array}{ccc} G_{-}\left(\mu \right) & \approx & \frac{-1}{{\left(\underset{\approx 0}{\underset{︸}{I_{1}}}+I^{*}+\underset{\approx 2\pi }{\underset{︸}{I_{2}}}\right)}^{2}}\frac{\partial I^{*}}{\partial \mu }\\ & \approx & \frac{\left|B\right|}{{\left[\mu -(2\pi B-A)\right]}^{2}}\\ & \approx & \frac{\left|B\right|}{{\left(\mu -\mu^{*}\right)}^{2}}\;\;\;\;\text{,}\;\text{for}\;\mu <\mu^{*} \end{array}$$ (2)

$$\begin{array}{ccc} G_{+}\left(\mu \right) & = & \frac{-1}{I_{0}^{2}}\frac{\partial I_{0}}{\partial \mu }\\ & \approx & \frac{1}{2\pi }\sqrt{\frac{g_{2}}{\bar{\mu }}}\\ & = & \frac{\sqrt{g_{2}/4\pi^{2}}}{{\left[\mu -\left(W_{0}+W_{m}\left(V_{2}-W_{m}\right)\right)\right]}^{1/2}}\;\;\text{,}\;\text{for}\;\mu >\mu^{*}. \end{array}$$ (3)

For *μ* < μ*, the change in ISI is dominated by the change in $I^{*}$ (the segment from V* to *V*_2_), and $I_{1}$ and $I_{2}$ are roughly constant by comparison (see Fig 4C). This allows *G*_−_ to be reduced to Eq (2b), which diverges as *μ* → 2*πB* − *A*. Although *A* and *B* depend on *μ*, plugging in numerical values reveals that 2*πB* − *A* → μ* as *μ* → μ*. For *μ* > μ* the gain depends only on $I_{0}$ and is found to scale as $1/\sqrt{\bar{\mu }}$ from above, similarly to results for the simple QIF model [14], with a rescaled $\bar{\mu }$. In this case the gain diverges when $\bar{\mu }\to 0$ which occurs when *μ* = *W*_0_ + *W*_*m*_(*V*_2_ − *W*_*m*_)≃μ*, which is also very close to μ*. This shows that the gain scales as 1/(*μ* − μ*)^2^ from bellow and as 1/(*μ* − μ*)^1/2^ from above, and explains why the peak gain should be near *μ* = μ*.

**Fig 7:**
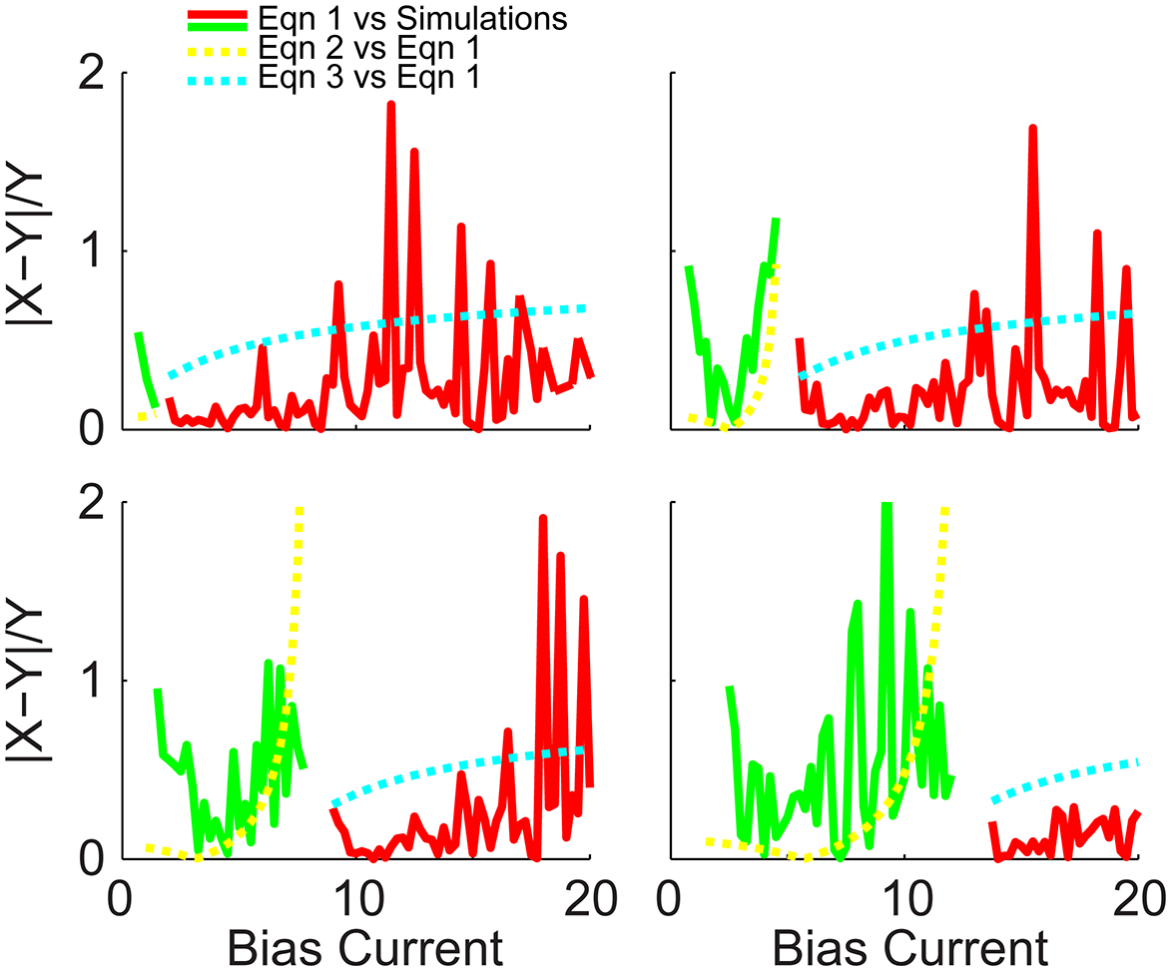
Comparison between results from the full theoretical and approximate gain equations. The quality of the full theoretical model is assessed by plotting the absolute difference between the derivative of Eq (1) and the numerical simulations, normalized by the simulated gain, plotted in green and red for *μ* < μ* and *μ* > μ* respectively. Additionally, the approximate gain Eqs (2) and (3) are compared to the derivative of Eq (1) with *τ*_*r*_ = 0, plotted in yellow and cyan dashed curves.

Eqs (2) and (3) are also superimposed in Fig 6, where the dashed yellow and cyan curves are approximations to the dashed green and red curves, and the solid green and red curves are approximations to the blue curve. The dashed yellow and green curves are in reasonable close agreement, and the solid green does capture the main effect of the boosting nonlinearity (i.e. increase in gain with increasing *μ*), however ignoring the initial spike in gain at the onset of spiking (blue curves). The solid red curves match the blue curves even better than the green curves (as there were fewer approximations needed). Although the dashed red and cyan curves do differ significantly, it is a roughly constant amount and the cyan curve still captures the essential scaling features. To quantify the quality of these approximations, the absolute differences of each pair, normalized by the target curve, is plotted in Fig 7. Red and green compare Eq (1) with the simulations, and yellow and cyan compare Eqs (2) and (3) with the derivative of Eq (1).

### Theoretical firing rate for the QIF model with spike generated resets: convergent iterative predictions

Coming back to the more physiological QIF model with spike generated reset conditions, which reproduces closely the HH model’s boosting nonlinearity as well as its bifurcations through bursting, the reset values, *x*_*reset*_ and *C*_*reset*_, are no longer given. These reset values may be estimated via an iterative algorithm (see Models and Methods), which may converge to a stable sequence of reset values. In the high bias region where the slow gating variable approximation is valid, a self-consistency condition can be used to generate successive gating variable reset values (and in turn ISIs) similarly to that of Richardson [15]. As described in Models and Methods, because *F*(*V*) > 0 in this regime, a result of the Fokker-Plank equation can be used to give the probability distribution of the voltage, *p*(*V*), which can be used to calculate *x*_*reset*_ = 〈*x*〉 and *C*_*reset*_ = 〈*C*〉 [15] according to Eqs (28)–(30). For low bias values where the slow gating variable approximation is not valid, however, Eq (30) no longer holds and the artificial action potential must be used to calculate new reset values.

**Fig 8:**
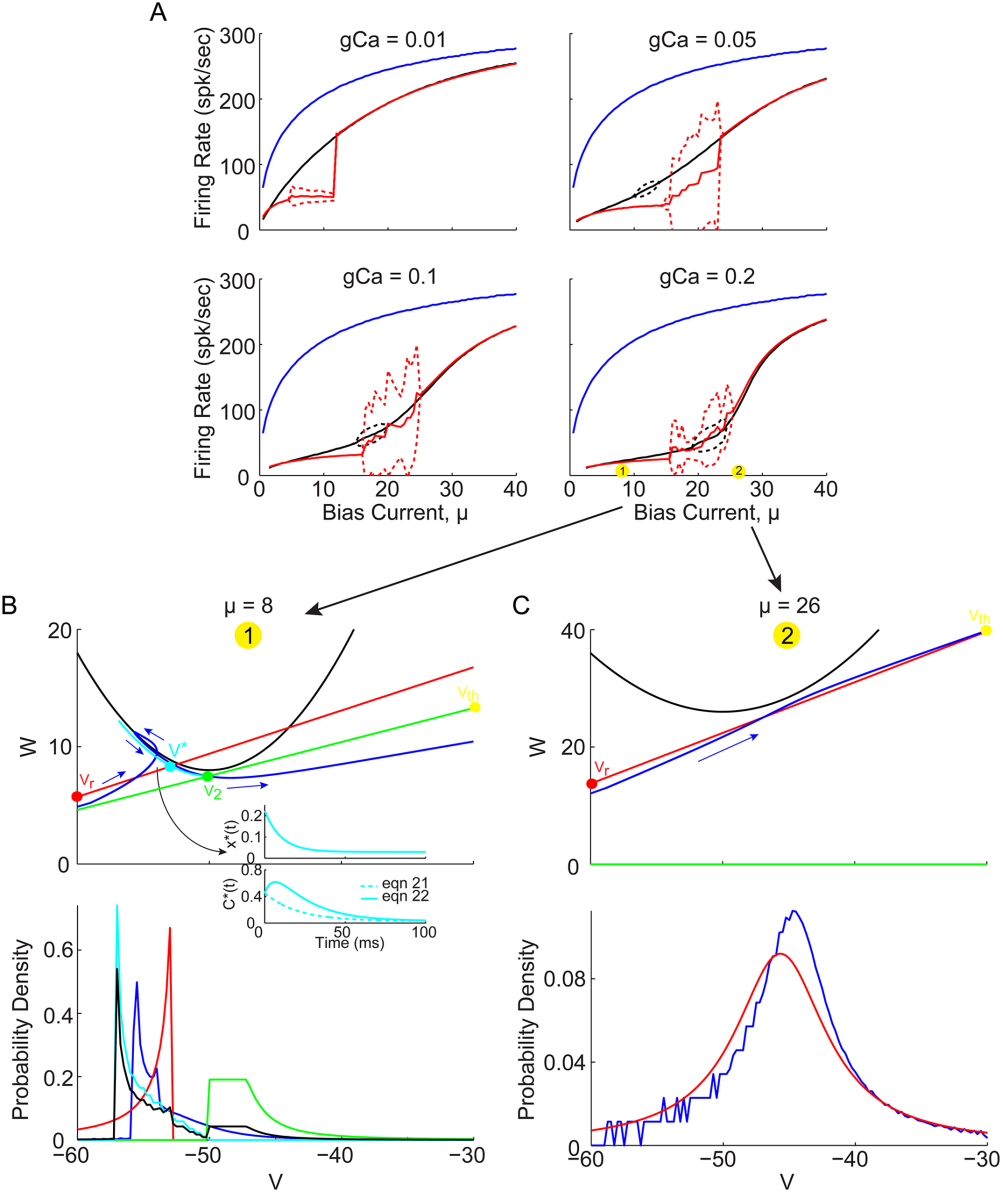
WV phase space projection for QIF model with spike waveform, and convergent iterative theoretical predictions for *μ* > μ* and *μ* → 0. (A) Firing rate for QIF with spike waveform, as in Fig 3A, in solid black lines for *g*_*Ca*_ > 0 (as indicated), and blue curves for *g*_*Ca*_ = 0. Iteratively estimated theoretical predictions (see Models and Methods for details) are superimposed in red, with solid lines indicating the mean 1/ISI, and dashed lines indicating standard deviation (SD) of 1/ISIs (the bursting patterns will be considered in the next figure). Theory shows excellent agreement above the bursting region where *F*(*V*) > 0, and reasonable agreement at very low bias currents. Two example bias currents are indicated by numbered yellow circles in the bottom right panel. (B) Low bias example trajectory in VW phase space. Simulated trajectory in blue (with direction of flow indicated by blue arrows), with theoretical trajectory connecting *V*_*reset*_ to V* (red line), then V* to *V*_2_ (cyan line), and finally *V*_2_ to *V*_*th*_ (green line). Inset shows the cyan trajectory in terms of decaying variables x*(*t*) and C*(*t*), and how Eq (22) captures the initial increase and then decrease in the calcium concentration, while Eq (21) does not. Below, the corresponding voltage probability density for the simulated trajectory (blue), and each of the red, cyan, and green segments (independently normalized), as well as their weighted combination (black, see Models and Methods). (C) Same as panel B, but for the high bias example point.

Letting the algorithm iterate, it may converge to a sequence of identical ISIs (i.e. stable 1-spk limit cycle), a sequence of 2 or more ISIs which repeats (i.e. stable 2- or 3-spk limit cycle; bursting), or even a sequence of ISIs that has no repeating patterns. After 20 iterations of transient ISIs, convergence has generally been reached and the mean and standard deviation of the subsequent sequences of 1/ISIs was used to estimates the f-I curves, as plotted in Fig 8A. The iterative theory can be seen to converge to stable 1-spk limit cycles in the limits of low and high bias current, as well as produce variable ISI sequences (red dashed) near the bursting in the QIF model (black dashed). The best agreement is actually achieved for the highest value of *g*_*Ca*_ = 0.2 in the bottom right panel of Fig 8A, where two example bias currents are indicated by yellow dots, which will be considered in the VW phase space below.

For the stable 1-spike limit cycles in reasonable agreement with the QIF simulations, the low and high bias example trajectories are plotted in the VW phase space in Fig 8B and 8C. These two trajectories can be seen to have the same fundamental geometry of those in Fig 4: in the high bias region the trajectories do not encounter the parabola, *μ* + *ψ*, while for low bias currents, they do. However, in Fig 8B the simulated QIF trajectory (blue curve) crosses the black parabola and changes direction in V, before crossing the parabola again and crossing back over itself (which is only possible since this is really a 3D phase space projected into 2D) before increasing to threshold. It is this trajectory that result in the specific AHP shape in which the voltage changes directions twice. In this case Eq (22) must be used to estimate the calcium decay from V* to *V*_2_, which is compared to that calculated analytically via Eq (21) in the Fig 8B inset (see Models and Methods for details). Once the time interval for each separate component is calculated as described in Models and Methods, the voltage distribution during each segment can be estimated and in the bottom of 8B can be seen to provide a not very accurate match to the simulation (compare black and blue curves), but it does have the marked features of the combined red and cyan peaks. These distributions in 8B, however, are not used in the iterative algorithm. For example 2 in the high bias regime (Fig 8C), both *V* and *W* can be seen to increase monotonically from reset to threshold, and the resulting voltage distribution is in much better agreement with the simulations.

**Fig 9:**
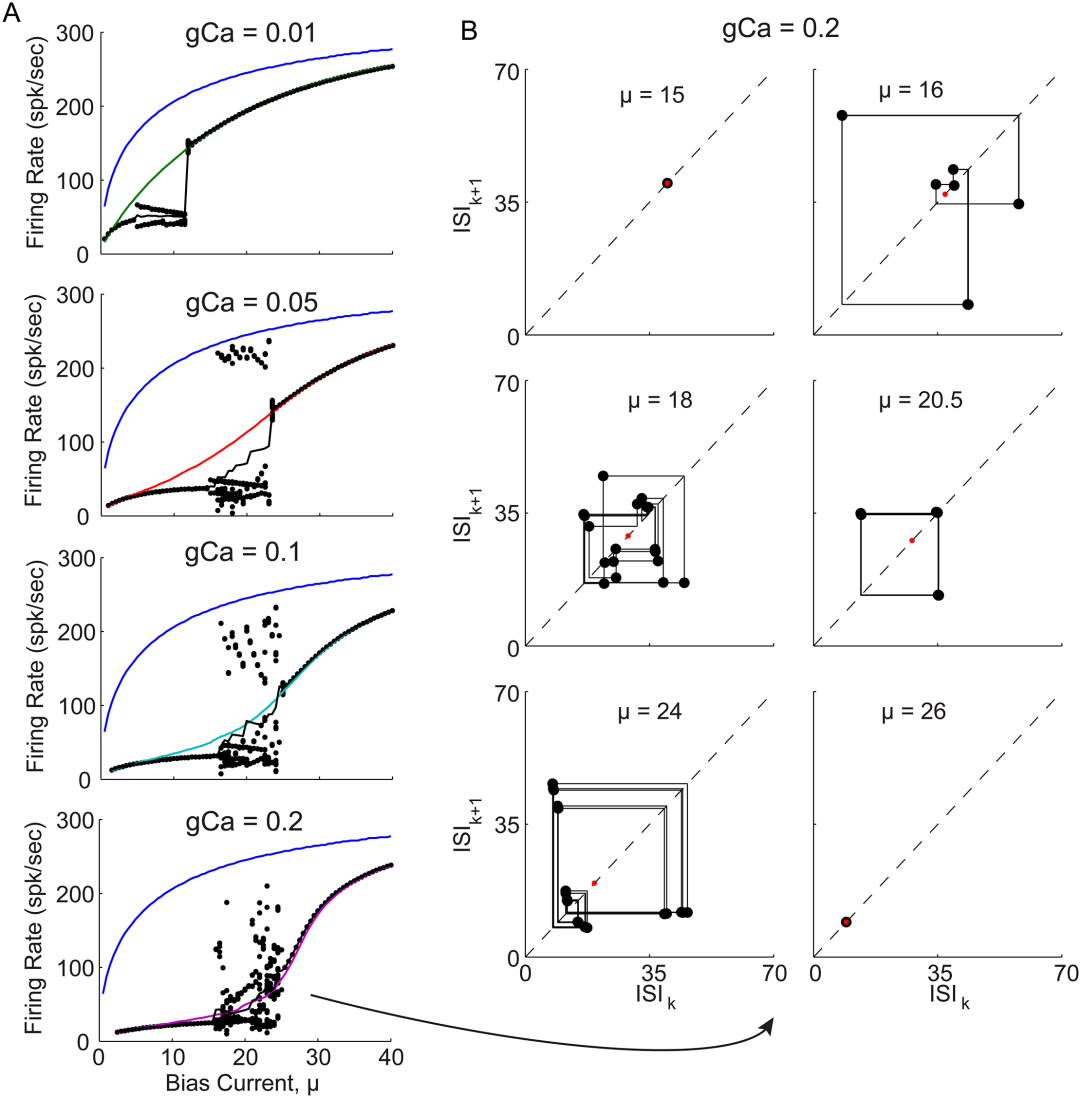
Iterative theoretical predictions for QIF model with spike waveform: stable and unstable 1-spike limit cycles. (A) f-I curves for QIF model with spike waveform, each panel comparing *g*_*Ca*_ = 0 (blue) with *g*_*Ca*_ > 0 as indicated (color), as well as the theoretical predictions (black). The iterative theory produces a sequence of ISIs, the final 20 are plotted as 1/ISI at each bias (black dots), and their average value versus bias is superimposed (solid black). (B) For the highest *g*_*Ca*_ value, ISI return maps are shown for six illustrative bias current values starting with a low bias stable 1-spk limit cycle (top left panel), through bifurcations to aperiodic spiking, with windows of repeating sequences, and back to stable 1-spk limit cycles at high biases (bottom right panel). The same 20 ISIs from A are also plotted in B. It can be seen that at *μ* = 20.5, a stable sequence of 3 intervals repeats. Similarly, the bottom left panel shows similar sequence of 3 ISIs almost repeats, but 2+ slightly different versions of it repeat, illustrating how regions with stable N-spike ISI sequences transition to other regions with stable M-spike ISI sequences.

Although the iterative theory captures the essential phase space geometry to explain the boosting nonlinearity, the results in the regions of bursting are considered next. In Fig 9 (left) the FI curves of the QIF with spike are again plotted, but now with each 1/ISI in the sequences (black dots). Clearly these do not follow the ordered period adding bifurcations see in Figs 1E and 2E. Although there does appear to be a window of order with a stable 3-spike burst (see Fig 9 right), this 3-spike sequence does not have the same structure as the bursts in Figs 1 and 2 (i.e. short-short-long ISI sequences) and may instead be considered an alternation of 1-spk and 2-spk bursts. While the iterative theoretical predictions do capture the low to high gain transition across the boosting nonlinearity, they do fail entirely at capturing the period adding bifurcations through bursting.

## Conclusions

To summarize, in this paper it was shown that a conductance-based Hodgkin-Huxley type vestibular neuron model with high voltage-activated calcium and calcium-activated potassium currents, can exhibit a boosting nonlinearity for increased calcium conductance, *g*_*Ca*_. In addition, the model exhibits a period adding bifurcation through bursting for intermediate bias currents separating the low and high gain regions of the boosting nonlinearity, with an AHP in the low gain region. In order to isolate the mechanism underlying the boosting nonlinearity, the HH model was reduced to a generalized QIF model that preserves the subthreshold bifurcation structure. With an artificial action potential to activate the gating variables, the QIF model reproduces the boosting nonlinearity and the bifurcation through bursting and AHP. To further simplify the model and tease apart the necessary ingredients for a boosting nonlinearity, QIF models were created that use fixed values for, Δ*x* and Δ*C*, and finally for, *x*_*reset*_ and *C*_*reset*_. For this simplified QIF a slow gating variable approximation was used as a starting point to derive an analytic equation for the f-I curves, and approximate expressions for the gain (i.e. its slope), showing the gain to be peaked at *μ* = μ*. An intuitive geometrical picture of how the trajectories through VW phase space shows that they differ qualitatively in the low and high bias regions of the boosting nonlinearity, and that these two types of trajectories provide the basis for understanding the boosting nonlinearity and deriving an expression for μ*. Finally, in the case of spike generated reset conditions, it is shown that an iterative algorithm can find stable 1-spk limit cycles that provide reasonable agreement in the limit that *μ* → 0, and excellent agreement in the high bias regime.

### Comparison to other two- and three-variable adaptive models

Previously a two-variable adaptive QIF model was studied and found to exhibit a similar boosting nonlinearity [14]. This model used a fixed reset value for its adaptation current, *W*, such that it would be reset *above* the parabolic function *μ* + *ψ*(*V*) for low bias, causing the voltage to initially move in the negative direction until it can cross below *ψ* and begin moving positively, towards threshold. Once *μ* is increased such that it is greater than *W*_*reset*_, the trajectories are then reset below *ψ* and increase monotonically towards threshold. This is a very similar mechanism of boosting, whereby spiking trajectories in the low gain region must cross (or come very close to) the V-nullcline, W* = *ψ*(V*), while trajectories in the high gain region do not. This mechanism also results in an AHP in the low gain region, where the voltage initially decreases through a slow minimum, but only changing directions once. However, because phase space trajectories can not cross over themselves, the low gain region only emerges when the reset conditions start the trajectory above *ψ*. In the three-variable adaptive QIF model considered here, the projections in the VW plane can cross themselves only because they have an extra dimension due to the *x* and *C* variables, and *W*(*x*, *C*). This allows trajectories in the QIF model to start below the V-nullcline, cross up above it, loop back below, cross themselves and off towards threshold. This is what gives my 3-variable QIF model’s spikes their signature AHP shape which initially increases before decreasing, unlike two-variable models.

Although the two-variable QIF model of Shlizerman and Holmes does not burst [14], its close relative the adaptive exponential-IF (aEIF, also two-variable) can produce bursting [11, 12], when the adaptation variable reset condition is instead *W*_*reset*_ = *W*(*t*_*spk*_) + Δ*W*. This produces bursting in a similar way as the adaptive QIF model: multiple short ISIs occur (which do not intersect *ψ*) with *W* increasing each time, until *W* has accumulated enough that the trajectory does intersect *ψ* and a long ISI occurs, terminating the burst. Although the exponential function in the aEIF model changes the shape of *ψ*, it does not change the basic concave-up geometry captured by the QIF. As such, it may be expected that the regions of such aEIF models that produce bursts might also indicate the presence of a boosting nonlinearity, however this has not been reported [11, 12]. In addition, although the aEIF model generally uses a simpler linear equation for the dynamics of the adaptation variable such as $\dot{W}=\left[a\left(V-V_{w}\right)-W\right]/\tau_{w}$, it still requires the further simplification that *a* = 0, to compute that 〈*W*〉 = Δ*W* a priori and apply a slow gating variable assumption for an analytic solution [10, 16].

Finally, Richardson analyzes a three-variable adaptive model very similar to ours [15], with spike-triggered calcium and calcium activated potassium, however he uses an artificial action potential with *V*_*max*_ = 0, that decays linearly to the reset *V*_*reset*_. He also avoids the problem of not knowing the value of 〈*W*〉 by using the slow gating variable assumption and self-consistency criterion to find it iteratively. He does not report a boosting nonlinearity or bursting, but remains in a region of parameter space where *F*(*V*) > 0 and no AHP [15]. In addition, different spike shapes with the QIF did have a significant effect on whether or not the adaptation currents were strong enough to produce either boosting or bursting, which is one possible explanation for our differing results. However, different values for the conductances *g*_*Ca*_ and *g*_*KCa*_ are used as well. Although the goal of this study was to understand the mechanisms that produce boosting in the HH model, the ultimate goal is to relate it back to experimental data from the vestibular system, and how it might be functionally relevant.

### Correspondence to vestibular nuclei neuron data

The HH model used in this paper is already a simplified version of the original vestibular nuclei neuron model developed by Av-Ron et al. [5], where only the ion channels necessary to generate the boosting nonlinearity were included. These channels were originally tuned to produce the characteristic bi-directional AHP that goes up and then down before rising to threshold (switching direction twice). This very AHP appears to be a signature that the model would likely produce a boosting nonlinearity (and bursting) if driven to sufficiently high bias currents that the AHP no longer occurs. However, this model was originally developed for *in vitro* preparations where the average baseline firing rate is much lower (i.e. ∼30–50*spk*/*s*) than in alert behaving animals (i.e. ∼60–80*spk*/*s*) [6]. This may be why such a boosting nonlinearity has not yet been observed *in vitro*. One would expect that if the *in vitro* recordings used current injections large enough that the AHP could no longer occur, that this would also be sufficiently large to reveal a boosting nonlinearity as well, an experimentally testable prediction of this manuscript.

It is also important that neurons are considerably more variable *in vivo*, requiring an additive noise term in the model [6]. Including such increased noise, simulations of the HH model still show the boosting nonlinearity, while the noise is sufficient to disrupt the bursting (not shown). Furthermore, analysis of the data in Massot et al. [4] has shown no evidence of bursting *in vivo*. To further improve the correspondence between the HH model and VN neurons, additive noise could be added to provide the appropriate coefficient-of-variation of the spontaneous spiking activity [4]. However, it is known that different noise intensities may be needed during spontaneous and driven stimulation conditions, as was found for vestibular afferent models [17]. Experimental efforts should therefore be made to measure both the mean firing rate and its variance as functions of bias current, using different stimuli, to further constrain accurate VN neuron models.

### Implications for sensory information processing in the vestibular system

The boosting nonlinearity was originally found in vestibular-only (VO) neurons in VN using narrow band noise stimuli with low (0–5 Hz) and/or high (15–20 Hz) frequency content, and it was found that when presented together the high frequency stimuli masked the response to low frequency stimuli [4]. A linear-nonlinear (LN) cascade model of the data could explain this masking effect and predict the % attenuation for additional stimuli. The statistics of naturally occurring head movements in primates have since been recorded [18] and indeed been found to have significantly higher power over the low frequency range than the high frequency range, making it unclear whether such masking would occur under natural conditions. This could be explored in a model using stimuli with naturalistic frequency content combined with afferent filters. Additionally, natural stimuli have combinations of angular and linear movements, which could also lead to masking between different axes of motion, rather than different frequency bands within one axis of motion.

It is well known that when neurons are driven across a common rectifying nonlinearity, it can result in increased spiking precision, with information lost about the stimuli in the zero gain region of the nonlinearity which also has a firing rate of zero. It is therefore possible that the boosting nonlinearity could allow the same increased spiking precision, potentially indicative of temporal encoding, to coexist with a standard rate coding since the low gain region still has non-zero gain and firing rate. Further studies with this model could therefore investigate the possibility of simultaneous rate and temporal coding, under natural stimulus conditions.

Finally, it should be pointed out that VO neurons are known to respond robustly to passively applied stimuli (i.e. head movements externally generated by the experimenter), but to show ∼70% to the self-generated stimuli studied [19], and that the large majority of natural stimuli recorded by Schneider et al. was indeed self-generated [18]. This suggests a potential role for the boosting nonlinearity: if self-generated stimuli elicit responses that are not sufficiently attenuated and cross the boosting nonlinearity, an increased level of spiking precision (or population synchrony) could signal a potential problem, without entirely disrupting the linearly decodable information remaining about the stimulus in the firing rate.

## Models and Methods

### Full HH model

The Hodgkin-Huxley (HH) type model of a VN neuron developed by Av-Ron et al. [5] and adapted by Schneider et al. [6] is studied in this paper. Specifically, the model includes spiking sodium and potassium currents governed by the single activation variable, *n* (as in the Morris-Lecar model), as well as a voltage-activated calcium current and calcium-activated potassium current, each governed by the activation variables, *x* and *C*, respectively. The additional calcium current, is activated by high voltages that occur during an action potential, and serves primarily to let calcium into the cell with only a small effect on membrane voltage. The additional potassium current, however, is only activated by the calcium that enters the cell when it spikes, and serves to reduce the voltage and prevent spiking. The additional persistent sodium and hyperpolarization-activated currents present in [5, 6] have been removed, as they are not necessary for the model to generate the boosting nonlinearity being investigated. This results in a 4-dimensional spiking neuron model governed by the following differential equations:

$$\begin{array}{ccc} C_{m}\frac{dV}{dt} & = & H_{1}\left(V,n,x,C\right)=\mu -I_{ions}\left(V,n,x,C\right)\\ \frac{dn}{dt} & = & H_{2}\left(V,n\right)=\left[n_{\infty }\left(V\right)-n\right]/\tau_{n}\\ \frac{dx}{dt} & = & H_{3}\left(V,x\right)=\left[x_{\infty }\left(V\right)-x\right]/\tau_{x}\\ \frac{dC}{dt} & = & H_{4}\left(V,x,C\right)=\left[C_{\infty }\left(V,x\right)-C\right]/\tau_{C}. \end{array}$$ (4)

*I*_*ions*_ = *I*_*Na*_ + *I*_*K*_ + *I*_*leak*_ + *I*_*Ca*_ + *I*_*KCa*_, with $C_{\infty }=-\frac{K}{R}I_{Ca}$, and the currents are given by the following additional equations:

$$\begin{array}{ccc} I_{Na}\left(V,n\right) & = & g_{Na}m_{\infty }^{3}\left(1-n\right)\left(V-V_{Na}\right)\\ I_{K}\left(V,n\right) & = & g_{K}n^{4}\left(V-V_{K}\right)\\ I_{leak}\left(V\right) & = & g_{L}\left(V-V_{L}\right)\\ I_{Ca}\left(V,x\right) & = & g_{Ca}x^{2}\left(V-V_{Ca}\right)\\ I_{KCa}\left(V,C\right) & = & g_{KCa}\frac{C}{C+K_{d}}\left(V-V_{K}\right), \end{array}$$ (5)

where the steady state activation variables obey the following equation: $z_{\infty }\left(V\right)=1/\left[1+\exp\left[-2a^{\left(z\right)}\left(V-V_{1/2}^{\left(z\right)}\right)\right]\right]$, for *z* ∈ {*n*, *x*}. All parameters are as used by Schneider et al. [6], unless otherwise stated. The calcium current equation *I*_*Ca*_ has also been modified from Schneider et al. to remove the calcium saturation term, $\frac{K_{r}}{C+K_{r}}$, to further simplify the model while preserving the boosting nonlinearity.

The fixed points (FPs) of the HH model can be found by setting the equations *H*_1_ = *H*_2_ = *H*_3_ = *H*_4_ = 0, then solving for, n* = *n*_∞_(V*), x* = *x*_∞_(V*), C* = *C*_∞_(V*), while V* must be found by plugging these into *H*_1_, and numerically finding the zeros of

$$\begin{array}{ccc} H_{1}\left(V,n^{*},x^{*},C^{*}\right) & = & \mu -g_{L}\left(V-V_{L}\right)-g_{Na}m_{\infty }^{3}\left(V\right)\left(1-n^{*}\left(V\right)\right)\left(V-V_{Na}\right)\\ & & -g_{K}n^{*4}\left(V\right)\left(V-V_{K}\right)\\ & & -g_{Ca}x^{*2}\left(V\right)\left(V-V_{Ca}\right)-g_{KCa}\frac{C^{*}\left(V\right)}{K_{d}+C^{*}\left(V\right)}\left(V-V_{K}\right), \end{array}$$ (6)

for a range of bias current values, *μ*. The stability of the fixed points can then be found by looking at the eigenvalues of the following matrix

$$\begin{array}{c} L_{HH}=(\begin{array}{cccc} \partial H_{1}/\partial V & \partial H_{1}/\partial n & \partial H_{1}/\partial x & \partial H_{1}/\partial C\\ \partial H_{2}/\partial V & \partial H_{2}/\partial n & 0 & 0\\ \partial H_{3}/\partial V & 0 & \partial H_{3}/\partial x & 0\\ \partial H_{4}/\partial V & 0 & \partial H_{4}/\partial x & \partial H_{4}/\partial C \end{array}), \end{array}$$ (7)

where the FP is stable if all its eigenvalues have negative real parts.

Setting *g*_*Ca*_ = 0 (and in turn *C* = 0), it is well known that *H*_1_(*V*) has a cubic form (or sideways ‘S’ shape), with a local minimum at a lower voltage and a local maximum at higher voltage. This shape does not change, but is translated vertically with changes in the bias current, *μ*. For sufficiently low values of *μ* there are three fixed points, only that with the lowest voltage is stable, and corresponds to the steady state resting potential. As *μ* is increased, the two fixed points at lower voltages annihilate in a saddle-node bifurcation at which point there is no stable fixed point, and the model generates action potentials via a stable limit cycle. It is also possible (when *g*_*Ca*_ > 0) for the lowest voltage fixed point to lose stability via a Hopf bifurcation. In this case the spiking limit cycle can coexist with all three unstable fixed points, with the two lower voltage fixed points annihilating at yet higher values of *μ*.

### Simplified QIF model

In order to find an analytic equation explaining the change in gain of the boosting nonlinearity, a reduced integrate-and-fire (IF) type model is used, that preserves the FP bifurcation structure of the HH model. This is done by removing the gating variable, *n*, and replacing the currents, *I*_*L*_(*V*) + *I*_*Na*_(*V*) + *I*_*K*_(*V*), with a nonlinear function *ψ*(*V*), and a voltage threshold and reset. Although simple constant or linear functions can be used for *ψ*(*V*), a concave up function is needed to reproduce the second bifurcation of two subthreshold fixed points in the case of the Hopf bifurcation at spiking onset. The simplest of these functions is the quadratic, *ψ*(*V*) = *g*_2_(*V* − *V*_2_)^2^, resulting in the generalized QIF model, governed by 3 differential equations:

$$\begin{array}{ccc} C_{m}\frac{dV}{dt} & = & F_{1}\left(V,x,C\right)=\mu +\psi \left(V\right)-I_{Ca}\left(V,x\right)-I_{KCa}\left(V,C\right)\\ \frac{dx}{dt} & = & F_{2}\left(V,x\right)=\left[x_{\infty }\left(V\right)-x\right]/\tau_{x}\\ \frac{dC}{dt} & = & F_{3}\left(V,x,C\right)=\left[C_{\infty }\left(V,x\right)-C\right]/\tau_{C}, \end{array}$$ (8)

where the only new parameters to define are *g*_2_ and *V*_2_.

In addition, the QIF model requires a boundary condition such that when the voltage reaches a threshold, *V*_*th*_, a spike is said to have occurred, and the voltage is returned to a reset value, *V*_*reset*_, for an absolute refractory period, *τ*_*r*_. However, because the high voltages occurring during the action potential are needed to drive the voltage-activated calcium currents, a simple piece-wise linear function, *V*_*spk*_(*t*), is used during the refractory period *t*_*spk*_ < *t* < *τ*_*r*_ (similar to Richardson [15]). The spike shape rises linearly to a maximum, and then decays linearly to the reset voltage according to

$$\begin{array}{ccc} V_{spk}\left(t\right) & = & V_{th}+\frac{V_{max}-V_{th}}{t_{1}}t\text{,}\;\text{.}\;\text{.}\;\text{.}\;\text{.}\;\text{.}\;\text{.}\;\text{for}\;0\leq t<t_{1}\\ & & V_{max}+\frac{V_{r}-V_{max}}{\tau_{r}-t_{1}}\left(t-t_{1}\right)\text{,}\;\text{.}\;\text{.for}\;t_{1}\leq t<\tau_{r} \end{array}$$ (9)

where *t* = 0 corresponds to the spike times. In this paper, the spike shape parameters, *V*_*max*_ = 30 mV, *t*_1_ = 0.4 ms, and *τ*_*r*_ = 3 ms are used. This results in the *x* and *C* gating variables changing according to *x*(*t*_*spk*_) → *x*(*τ*_*r*_) = *x*(*t*_*spk*_) + Δ*x*, and *C*(*t*_*spk*_) → *C*(*τ*_*r*_) = *C*(*t*_*spk*_) + Δ*C*, where Δ*x* and Δ*C* are calculated by plugging Eq (9) into Eq (8b) and (8c) and numerically integrating *x*(*t*) and *C*(*t*) from *t*_*spk*_ to *τ*_*ref*_.

The QIF model can be further simplified by removing *V*_*spk*_(*t*) and using either fixed Δ*x* and Δ*C*, or fixed *x*_*reset*_ and *C*_*reset*_, resulting in *x*(*t*_*spk*_ + *τ*_*r*_) = *x*_*reset*_ and *C*(*t*_*spk*_ + *τ*_*r*_) = *C*_*reset*_. This results in two more parameters, either *x*_*reset*_ and *C*_*reset*_, or Δ*x* and Δ*C*, which must be defined, instead of *V*_*max*_ and *t*_1_.

The fixed points (FPs) of this simplified QIF model do not depend on the artificial spike shape or reset boundary conditions, and can be found by setting the equations *F*_1_ = *F*_2_ = *F*_3_ = 0, and solving for, x* = *x*_∞_(V*), C* = *C*_∞_(V*), as before, with V* now being found by plugging these into *F*_1_, and numerically finding the zeros of

$$\begin{array}{ccc} F_{1}\left(V,x^{*},C^{*}\right) & = & \mu -g_{2}\left(V-V_{2}\right)-g_{Ca}x^{*2}\left(V\right)\left(V-V_{Ca}\right)-g_{KCa}\frac{C^{*}\left(V\right)}{K_{d}+C^{*}\left(V\right)}\left(V-V_{K}\right)\\ & = & 0. \end{array}$$ (10)

The stability of the fixed points can then be found by looking at the eigenvalues of the following matrix

$$\begin{array}{c} L_{QIF}=(\begin{array}{ccc} \partial F_{1}/\partial V & \partial F_{1}/\partial x & \partial F_{1}/\partial C\\ \partial F_{2}/\partial V & \partial F_{2}/\partial x & 0\\ \partial F_{3}/\partial V & \partial F_{3}/\partial x & \partial F_{3}/\partial C \end{array}). \end{array}$$ (11)

Although the subthreshold fixed points and their stability depend only on the system of Eq (8), the reset values, *x*_*reset*_ and *C*_*reset*_, behave as additional bifurcation parameters, similarly to the reset parameters in the adaptive two-variable models studied by Naud et al. [12].

To estimate the QIF model parameters *g*_2_ and *V*_2_ from the HH model, *g*_*Ca*_ can be set to zero, and the nonlinear functions in *H*_1_(*V*, n*) expanded to second order in *V*, around its approximate minimum (≈ −50 mV, seen by plotting),

$$\begin{array}{ccc} H_{1}\left(V\right) & = & \mu -g_{L}\left(V-V_{L}\right)-g_{Na}\underset{\approx a_{1}+b_{1}\left(V+50\right)}{\underset{︸}{m_{\infty }^{3}\left(V\right)}}\left(1-\underset{\approx a_{2}+b_{2}\left(V+50\right)}{\underset{︸}{n_{\infty }\left(V\right)}}\right)\left(V-V_{Na}\right)\\ & - & g_{K}\underset{\approx a_{3}+b_{3}\left(V+50\right)}{\underset{︸}{n_{\infty }^{4}\left(V\right)}}\left(V-V_{K}\right),\\ & \approx & \mu +k+a{\left(V-h\right)}^{2}, \end{array}$$ (12)

with $a_{1}=m_{\infty }^{3}\left(V=-50\right)$, *a*_2_ = *n*_∞_(*V* = −50), $a_{3}=n_{\infty }^{4}\left(V=-50\right)$, $b_{1}=\partial m_{\infty }^{3}/\partial V\left(V=-50\right)$, *a*_2_ = ∂*n*_∞_/∂*V*(*V* = −50), and $a_{3}=\partial n_{\infty }^{4}/\partial V\left(V=-50\right)$. Solving for a and h can then be used to estimate *g*_2_ and *V*_2_, however, the values *g*_2_ = 0.1 and *V*_2_ = −50 mV do a sufficient job to reproduce the HH model’s features of interest.

A complete solution of this model would result in *V*(*t*), *x*(*t*), and *C*(*t*), and can result in tonic firing of a single repeated interspike-interval (ISI), or bursts of two or more ISIs in a sequence which repeats, as well as possibly aperiodic spiking with sequences of ISIs which never repeat. In the entire 3D phase space, there is a single trajectory deterministically connecting *V*_*r*_ to *V*_*th*_, for each possible combination of *x*_*reset*_ and *C*_*reset*_ values which occur at the voltage reset. The trajectories cannot intersect and the entire phase space of trajectories is defined by the system of Eq (8), but the particular trajectory for each ISI is determined by the values of *V*_*reset*_, *x*_*reset*_, and *C*_*reset*_. The *x* and *C* values occurring at the voltage threshold may of course be different, and not necessarily result in the same reset values, and may therefore be reset onto a different nearby trajectory in phase space. In my simplified QIF with fixed *x*_*reset*_ and *C*_*reset*_ reset values, together with *V*_*reset*_, the voltage is reset onto the same trajectory after each spike, resulting in only tonic spiking of a single repeated ISI. In this case the ISI can be estimated analytically, with the values of *x*_*reset*_ and *C*_*reset*_ defined as model parameters.

### Slow gating variable approximation for fixed reset conditions

The additional gating variables, *x* and *C*, have time constants of 10 and 20 ms, compared to the average membrane time constant of ≈2 ms, and can thus be assumed to vary slowly by comparison (i.e. $\dot{x}\approx \dot{C}⪡\dot{V}$). Although the gating variables may be reset instantaneously during the refractory period following spiking, this approximation only needs hold from the end of the refractory period until the next spike. Additionally subthreshold regions in which this approximation breaks down, such as during an AHP, will be identified and dealt with separately. This assumption allows the gating variables to be approximated by their initial values, *x* ≈ *x*_*reset*_ and *C* ≈ *C*_*reset*_. As a result, the additional calcium related currents depend only on *V*, and can be defined in the adaptation current, $\bar{W}\left(V\right)$, as

$$\begin{array}{ccc} \bar{W}\left(V\right) & = & I_{Ca}\left(V,x_{r}\right)+I_{KCa}\left(V,C_{r}\right)\\ \bar{W}\left(V\right) & = & \underset{\equiv W_{m}}{\underset{︸}{\left(g_{Ca}x_{r}^{2}+g_{KCa}\frac{C_{r}}{C_{r}+K_{d}}\right)}}V\underset{\equiv W_{0}}{\underset{︸}{-(g_{Ca}x_{r}^{2}V_{Ca}+g_{KCa}\frac{C_{r}}{C_{r}+K_{d}}V_{K})}}\\ \bar{W}\left(V\right) & = & W_{0}+W_{m}V. \end{array}$$ (13)

$\bar{W}\left(V\right)$ is simply linear in *V*, always having a positive slope (except when *g*_*Ca*_ = 0, causing *W*_0_ = *W*_*m*_ = 0). This results in the system of Eqs in 8, reducing to a single differential equation

$$\begin{array}{c} \frac{dV}{dt}=F\left(V\right)=\mu +\psi \left(V\right)-\bar{W}\left(V\right), \end{array}$$ (14)

where *ψ*(*V*) > 0 represents the spike-generating currents which always drive the membrane voltage *towards* threshold, and $\bar{W}\left(V\right)>0$ represents the calcium and calcium-activated potassium currents which always act to drive the voltage *away* from threshold. It is because *V*_*K*_ < *V*_*r*_ ≤ *V* ≤ *V*_*th*_ < *V*_*Ca*_, that although the calcium current always serves to depolarize the membrane, the stronger calcium-activated potassium current always serves to hyperpolarize the cell.

In the approximate 1D system defined by Eq (14), *μ* + *ψ*(*V*) is a parabola with its minimum at *μ*, and $\bar{W}\left(V\right)$ is a line with positive slope, independent of *μ*. This gives two possible scenarios: for low enough *μ* the parabola and line intersect, while for high enough *μ* the parabola and the line do not intersect. If there is an intersection, then *F*(*V*) = 0 at that voltage, and the approximate 1D system should have a fixed point, but since the system is really a 3D system, it only indicates that the slow gating variable approximation breaks down. Although the initial conditions at voltage reset could correspond to a region where $\bar{W}>\psi$, as in [12, 14], this does not occur for the model parameters considered in this paper.

Since the parabola is translated linearly with *μ*, there must always exist a bias current, μ*, such that *F*(*V*) > *ϵ* for *μ* > μ*, where the parabola, *μ* + *ψ* − *ϵ* and the line, $\bar{W}$ intersect at a single point. The value of *ϵ* is chosen to be 0.5, small but non-zero, to avoid divergent calculations involving 1/*F*(*V*). For *g*_*Ca*_ > 0, the bias current μ*, can be defined by *F*(μ*, *V*) = *ϵ*:

$$\begin{array}{c} \mu^{*}=\frac{{\left(2g_{2}V_{2}+W_{m}\right)}^{2}}{4g_{2}}-g_{2}V_{2}^{2}+W_{0}+\epsilon . \end{array}$$ (15)

For *μ* > μ*, *F*(*V*) > *ϵ* and it is straightforward to integrate Eq (14) from reset to threshold to calculate the ISI (consider this case 1). But for *μ* < μ*, $\dot{V}<\epsilon$ for a range of *V* in which the slow gating variable assumption cannot be made and Eq (14) cannot be used (consider this case 2). It should be noted that in the limit that *g*_*Ca*_ → 0, μ* → *μ*, as expected.

**Case 1**: *μ* > μ* **and** $|\dot{V}|>\epsilon$ With *μ* > μ* and $|\dot{V}|>\epsilon$, the voltage moves monotonically from reset to threshold, and Eq (14) can be integrated to get the time interval

$$\begin{array}{c} I_{0}\left(\mu \right)=\int_{V_{r}}^{V_{th}}\frac{dV}{F(V)}. \end{array}$$ (16)

Plugging Eq (14) into 16 results in

$$\begin{array}{ccc} I_{0} & = & \int_{V_{r}}^{V_{th}}\frac{dV}{\bar{\mu }+g_{2}{\left(V-{\bar{V}}_{2}\right)}^{2}}\\ & = & \frac{1}{\sqrt{g_{2}\bar{\mu }}}\tan^{-1}[\frac{\sqrt{g_{2}\bar{\mu }}\left(V_{th}-V_{r}\right)}{\bar{\mu }+g_{2}\left(V_{th}-{\bar{V}}_{2}\right)\left(V_{r}-{\bar{V}}_{2}\right)}], \end{array}$$ (17)

where the new variables: $\bar{\mu }=\mu -W_{0}-W_{m}\left(V_{2}-W_{m}\right)$, ${\bar{V}}_{2}=V_{2}+W_{m}/2g_{2}$ have been defined. In the limit that *g*_*Ca*_ → 0, $\bar{\mu }\to \mu$ and ${\bar{V}}_{2}\to V_{2}$, and the solution to the to the simple QIF model is recovered [14]. Further letting *g*_2_ → 0, the well known IF model ISI, $I_{IF}=\left(V_{th}-V_{r}\right)/\mu$, results.

**Case 2**: *μ* < μ*

**Case 2a**: *V*_*r*_ ≤ *V* ≤ V*, **with** $\dot{V}>\epsilon$ For low bias currents, *μ* < μ*, there are two voltages at which the depolarizing current, *μ* + *ψ*(*V*) is balanced by the hyperpolarizing adaptation current, $\bar{W}\left(V\right)$, corresponding to fixed points where *F*(*V*) = 0. However, at the reset point, (*V*_*r*_, *W*_*r*_), *F*(*V*) is positive and remains so until the voltage reaches the *ϵ*-neighbourhood of the lower intersection point, V*, defined by

$$\begin{array}{c} V^{*}=\frac{2g_{2}V_{2}+W_{m}-\sqrt{{\left(2g_{2}V_{2}+W_{m}\right)}^{2}-4g_{2}\left(\mu -\epsilon +g_{2}V_{2}^{2}-W_{0}\right)}}{2g_{2}}, \end{array}$$ (18)

where *F*(V*) = *ϵ*. In this case, the voltage evolves according to Eq (14) from *V*_*r*_ up to V*, with $\bar{\mu }<0$ in this region, resulting in

$$\begin{array}{ccc} I_{1} & = & \int_{V_{r}}^{V_{-}^{*}}\frac{dV}{-|\bar{\mu }|+g_{2}{\left(V-{\bar{V}}_{2}\right)}^{2}}\\ & = & \frac{-1}{2\sqrt{g_{2}\left|\bar{\mu }\right|}}\ln|\frac{\left[1+\sqrt{g_{2}/\left|\bar{\mu }\right|}\left(V_{-}^{*}-{\bar{V}}_{2}\right)\right]\left[1-\sqrt{g_{2}/\left|\bar{\mu }\right|}\left(V_{r}-{\bar{V}}_{2}\right)\right]}{\left[1-\sqrt{g_{2}/\left|\bar{\mu }\right|}\left(V_{-}^{*}-{\bar{V}}_{2}\right)\right]\left[1+\sqrt{g_{2}/\left|\bar{\mu }\right|}\left(V_{r}-{\bar{V}}_{2}\right)\right]}|, \end{array}$$ (19)

with $\bar{\mu }$ and ${\bar{V}}_{2}$ defined as in Eq (17).

**Case 2b**: V* ≤ *V* ≤ *V*_2_, **with** $|\dot{V}|<\epsilon$ Once the membrane voltage has reached V*, *F*(*V*)≤*ϵ* and the slow gating variable approximation can no longer be made, and *x* and *C* must be allowed to evolve in time. It is assumed that at V* the gating variables result in an adaptation current W* > *μ*, and that they can now decay until the adaptation current, W*(*t*), reaches the bottom of the parabola at *μ* − *ϵ*. In this region, $\dot{V}\approx 0$ and $V\approx \left(V^{*}+V_{2}\right)/2\equiv {\bar{V}}^{*}$, so that one can solve *F*_2_ and *F*_3_ for x*(*t*) and C*(*t*). Assuming $x_{\infty }^{*}\equiv x_{\infty }\left({\bar{V}}^{*}\right)$, Eq (8b) gives

$$\begin{array}{c} x^{*}\left(t\right)=x_{\infty }^{*}-\left(x_{\infty }^{*}-x_{r}\right)e^{-t/\tau_{x}}, \end{array}$$ (20)

by requiring x*(*t* = 0) = *x*_*r*_. Now to solve Eq (8c), one should plug in x*(*t*), as calculated above, however to get an analytic solution, it is assumed that $x^{*}\left(t\right)\approx x_{\infty }^{*}$, and $C_{\infty }^{*}\equiv C_{\infty }\left(V^{*},x_{\infty }^{*}\right)$ is defined, resulting in

$$\begin{array}{c} C^{*}\left(t\right)=C_{\infty }^{*}-\left(C_{\infty }^{*}-C_{r}\right)e^{-t/\tau_{C}}. \end{array}$$ (21)

To get a more accurate prediction, Eq (8c) can be numerically integrated according to

$$\begin{array}{c} C_{2}^{*}\left(t_{i}\right)=C_{2}^{*}\left(t_{i-1}\right)+\Delta t\left[C_{\infty }\left(V^{*},x^{*}\left(t_{i-1}\right)\right)-C_{2}^{*}\left(t_{i-1}\right)\right]/\tau_{C}, \end{array}$$ (22)

with C*(*t*_0_) = *C*_*r*_, in either case.

The gating variable dynamics in turn cause changes in W*(*t*). It is then assumed that W*(*t*) decays until it reaches the bottom of the parabola *μ* + *ψ*(*V*) − *ϵ*, at *V*_2_. This time can then be solved for, W*(t*) = *μ* − *ϵ*,

$$\begin{array}{c} W^{*}\left(t^{*}\right)=g_{Ca}\underset{\approx a_{1}+b_{1}t^{*}}{\underset{︸}{x^{*}{\left(t^{*}\right)}^{2}}}\left(V^{*}-V_{Ca}\right)+g_{KCa}\underset{\approx a_{2}+b_{2}t^{*}}{\underset{︸}{\frac{C^{*}\left(t^{*}\right)}{C^{*}\left(t^{*}\right)+K_{d}}}}\left(V^{*}-V_{K}\right)=\mu -\epsilon \end{array}$$ (23)

by expanding to first order in time. The resulting coefficients are: $a_{1}=x_{r}^{2}$, *a*_2_ = *C*_*r*_/(*K*_*d*_ + *C*_*r*_), and

$$\begin{array}{ccc} b_{1} & = & \frac{\partial x^{*}{\left(t\right)}^{2}}{\partial t}{|}_{t=\tau_{x}}=\frac{2x^{*}\left(\tau_{x}\right)\left(x_{\infty }^{*}-x_{r}\right)}{\tau_{x}}e^{-1}\\ b_{2} & = & \frac{\partial }{\partial t}[\frac{C^{*}\left(t\right)}{K_{d}+C^{*}\left(t\right)}]{|}_{t=\tau_{C}}=\frac{K_{d}\left(C_{\infty }^{*}-C_{r}\right)}{{\left(K_{d}+C^{*}\left(\tau_{C}\right)\right)}^{2}\tau_{C}}e^{-1}. \end{array}$$ (24)

This allows t* to be found,

$$\begin{array}{ccc} I^{*} & = & t^{*}-0=\frac{\mu -\epsilon -[g_{Ca}a_{1}\left(V^{*}-V_{Ca}\right)+g_{KCa}a_{2}\left(V^{*}-V_{K}\right)]}{g_{Ca}b_{1}\left(V^{*}-V_{Ca}\right)+g_{KCa}b_{2}\left(V^{*}-V_{K}\right)}\\ & = & \frac{\mu -\epsilon -A}{B}, \end{array}$$ (25)

where *A* ≡ [*g*_*Ca*_ *a*_1_(V* − *V*_*Ca*_) + *g*_*KCa*_ *a*_2_(V* − *V*_*K*_)] and *B* ≡ *g*_*Ca*_ *b*_1_(V* − *V*_*Ca*_) + *g*_*KCa*_ *b*_2_(V* − *V*_*K*_). This shows that *A* represents the amount of adaptation current, *W*, when the voltage enters the $\dot{V}<\epsilon$ region at V*, while *B* represents rate of change of adaptation current, due to the decay of the gating variables *x* and *C*. This gives the simple geometric interpretation that the time interval $I^{*}$ is equal to the “distance” that *W* must travel, divided by the “velocity” at which *W* travels.

At this point in time, the adaptation current *W* has decayed to *W*(t*) = *μ* − *ϵ*, and *V*(t*) = *V*_2_, and the voltage is once again free to increase monotonically until threshold.

**Case 2c**: *V*_2_ ≤ *V* ≤ *V*_*th*_, **with** $\dot{V}>\epsilon$ For the remaining trajectory, I require a new adaptation current, ${\bar{W}}^{*}$, using the decayed gating variables instead of their initial reset values. However, I also require that ${\bar{W}}^{*}\left(V_{2}\right)=\mu -\epsilon$ to have the desired initial conditions, resulting in

$$\begin{array}{ccc} {\bar{W}}^{*}\left(V\right) & = & W_{0}^{*}+W_{m}^{*}V\\ W_{m}^{*} & = & g_{Ca}x^{*}{\left(t^{*}\right)}^{2}+g_{KCa}\frac{C^{*}\left(t^{*}\right)}{K_{d}+C^{*}\left(t^{*}\right)}\\ W_{0}^{*} & = & \mu -\epsilon -W_{m}^{*}V_{2}. \end{array}$$ (26)

Eq (14) can then be integrated from *V*_2_ to *V*_*th*_, resulting in

$$\begin{array}{ccc} I_{2} & = & \int_{V_{2}}^{V_{th}}\frac{dV}{{\bar{\mu }}^{*}+g_{2}{\left(V-{\bar{V}}_{2}^{*}\right)}^{2}}\\ & = & \frac{1}{\sqrt{g_{2}{\bar{\mu }}^{*}}}\tan^{-1}[\frac{\sqrt{g_{2}{\bar{\mu }}^{*}}\left(V_{th}-V_{2}\right)}{{\bar{\mu }}^{*}+g_{2}\left(V_{th}-{\bar{V}}_{2}^{*}\right)\left(V_{2}-{\bar{V}}_{2}^{*}\right)}], \end{array}$$ (27)

where the new variables: ${\bar{\mu }}^{*}=\mu -W_{0}^{*}-W_{m}^{*}\left(V_{2}-W_{m}^{*}\right)$, ${\bar{V}}_{2}^{*}=V_{2}+W_{m}^{*}/2g_{2}$ are again defined. Once the voltage has reached threshold, in this case the *x* and *C* variables have decayed from their reset values to new values at the occurrence of the new spike, *x*(*t*_*spk*_) = x*(t*) and *C*(*t*_*spk*_) = C*(t*). In this case, the fixed gating variable reset conditions are independent of these threshold values, but in the case of the spike generated resets, they will depend strongly on these threshold values.

### Iterative Theoretical Predictions: Stable and Unstable Limit Cycles

For the QIF model with spike generated reset conditions, the values of *x*_*reset*_ and *C*_*reset*_ are not known. However, with the theory described above, for fixed resets, there are two general types of ISI trajectories: Case 1, μ*(*x*_*reset*_, *C*_*reset*_) < *μ* in which $\dot{V}>\epsilon$ and the slow variable approximation holds, and case 2, μ*(*x*_*reset*_, *C*_*reset*_) > *μ* in which the slow variable approximation is violated and the gating variables are allowed to decay.

For case 1, *μ* > μ*, the slow gating variable assumption is that *x* ≈ 〈*x*〉 = *x*_*reset*_ and *C* ≈ 〈*C*〉 = *C*_*reset*_ are constant. In this case, the ISI is easily computed by Eq (17), and a simple result of the Fokker-Plank equation corresponding to Eq (14), gives the steady state voltage distribution

$$\begin{array}{c} p_{0}\left(V\right)=\frac{1}{F(V)}/\int_{V_{r}}^{V_{th}}\frac{dV}{F(V)}\text{,}\;\text{for}\;V_{r}<V<V_{th}, \end{array}$$ (28)

where the normalization constant is in fact the ISI, $I_{0}$. However, this is only the subthreshold voltage distribution, and doesn’t include the voltage distribution of the action potential, *p*_*spk*_(*V*), during the refractory period. To get the full voltage distribution these two distributions are combined, weighted according to their fraction of the total ISI:

$$\begin{array}{c} p\left(V\right)=A[\frac{I_{0}}{I_{0}+\tau_{r}}p_{0}\left(V\right)+\frac{\tau_{r}}{I_{0}+\tau_{r}}p_{spk}\left(V\right)], \end{array}$$ (29)

where *A* is a new normalization coefficient. This leads to the same gating variable self-consistency equations from Eq (5b) and (5c) as in [15]:

$$\begin{array}{ccc} \left\langle x\right\rangle & \approx & \int x_{\infty }\left(V\right)p\left(V\right)dV\\ \left\langle C\right\rangle & \approx & \int C_{\infty }\left(V,x_{\infty }\left(V\right)\right)p\left(V\right)dV. \end{array}$$ (30)

In this case the iterative algorithm outputs a voltage distribution, *p*(*V*), which is used to estimate the mean gating variables which will be used in the next iteration. If they again result in *μ* > μ*, the same procedure repeats, and may converge to a stable sequence of a single ISI. If not, the algorithm will proceed to case 2.

If the reset conditions result in case 2, *μ* < μ*, the gating variables will get a chance to decay, as estimated in Models and Methods. Therefore, because the slow gating variable approximation is violated, Eq (30) can no longer be used as the self-consistency criterion. Now the artificial action potential must be used to numerically reset the gating variables, and if the amount they decay is stabilized by the amount they are reset, a single trajectory and ISI will repeat. However, if they do not match, a new trajectory will be selected, which may again result in case 2, or take the algorithm back to case 1.

The algorithm may converge to a single repeated ISI, estimated either via case 1 in the high bias regime, or case 2 in the low bias regime, however, the algorithm may also result in a sequence of 2 or more ISIs which repeat periodically (i.e. bursting), or even an aperiodic sequence of ISIs which do not contain any repeating pattern. The sequences of ISIs and gating variable reset values, can be analyzed via the ISI return map, *ϕ*: *ISI*_*k*_ → *ISI*_*k*+1_, to quantify their stability.
